# Post-stroke sequelae risk score (PSSRS): a 16-year retrospective study developing an internally validated multi-algorithm model for post-stroke sequelae

**DOI:** 10.3389/fneur.2026.1833445

**Published:** 2026-10-15

**Authors:** Yiqun Dong, Rongji Lu, Yuying Zhang, Yuteng Huang, Ziyi Wang, Jiman Sun, Siyi Li, Yuxin Jia, Ying Tian, Haodong Hu, Xifeng Li, Yanchao Liu, Chuanzhi Duan

**Affiliations:** 1 Department of Cerebrovascular Surgery, Neurosurgery Center, The Neurosurgery Institute of Guangdong Province, Zhujiang Hospital, The National Key Clinical Specialty, Southern Medical University, Guangzhou, China; 2 School of Biomedical Engineering, Southern Medical University, Guangzhou, Guangdong, China; 3 School of Nursing, Southern Medical University, Guangzhou, Guangdong, China; 4 School of Mechanical Engineering, Dongguan University of Technology, Dongguan, China

**Keywords:** biomarkers, machine learning, post-stroke sequelae, precision medicine, prognostic model, risk prediction

## Abstract

**Introduction:**

Post-stroke sequelae (PSS) encompass persistent functional impairments after stroke and may benefit from early risk stratification. We developed and internally validated a time-to-event prediction model based on routinely available clinical and biochemical variables.

**Method:**

This retrospective single-center cohort included 2,095 patients hospitalized with acute stroke at Zhujiang Hospital between 2008 and 2024. Thirty-seven candidate variables were assessed. The cohort was split 7:3 into training (*n* = 1,467) and internal test (*n* = 628) sets before preprocessing, feature selection, tuning, and model comparison. Univariate Cox, multivariable Cox, least absolute shrinkage and selection operator (LASSO), and random survival forest (RSF) analyses identified eight intersection variables. A total of 101 survival-model pipelines were compared using training-set resampling; the selected model was then evaluated in the internal test set.

**Result:**

The StepCox (forward) + RSF pipeline showed the highest prespecified training-resampling performance. Its Harrell C-index was 0.87 in the training set and 0.85 in the internal test set. Test-set time-dependent AUROCs at 1, 3, and 5 years were 0.800 (95% CI, 0.760–0.840), 0.830 (0.790–0.870), and 0.820 (0.780–0.860), respectively; corresponding AUPRCs were 0.748, 0.772, and 0.761, and Brier scores were 0.140, 0.170, and 0.180. Calibration slopes were 0.96, 0.93, and 0.90. LDH, CK-MB, and DBIL were among the leading contributors in the model-interpretation analyses.

**Conclusion:**

PSSRS provides an internally validated framework for estimating individualized risk of post-stroke sequelae. External temporal and multicenter validation is required before clinical implementation.

## Introduction

Cerebral stroke is an acute cerebrovascular disease characterized by a group of conditions where cerebral tissue suffers damage due to insufficient blood supply caused by thrombus formation or hemorrhage resulting from ruptured cerebral vessels. According to World Health Organization statistics, approximately 15 million new cases of stroke occur globally each year (1). Endovascular thrombectomy (EVT) is an important treatment for eligible patients with acute ischemic stroke; however, the present cohort comprised patients hospitalized with acute stroke and was not restricted to EVT recipients. Post-stroke sequelae (PSS) may occur across treatment pathways (2, 41). Globally, 93.816 million stroke survivors currently endure long-term sequelae, experiencing significantly diminished quality of life (3). Consequently, maintaining favorable long-term outcomes and minimizing severe long-term complications have become fundamental objectives in stroke patient management. Among post-stroke patients, PSS not only causes long-term impairment in cognitive, motor, and language functions but also significantly increases disability rates and complication risks, severely impacting patients’ quality of life and exacerbating the social and medical burden (4, 43). However, research on the occurrence of PSS remains limited. Some stroke studies primarily focus on predicting stroke risk in sub-healthy populations (45). Thus, timely identification of high-risk factors for PSS and implementation of effective preventive and therapeutic measures are crucial for improving PSS prognosis (5).

The occurrence of PSS is closely associated with irreversible brain tissue damage. Its etiology is complex, manifesting clinically as physical dysfunction, cognitive and mental decline, and a range of complication risks. It significantly diminishes patients’ quality of life and triggers multiple social and economic harms (4). The mechanisms underlying PSS remain unclear. It is generally believed that multiple pathophysiological processes collectively contribute to its development and progression. These include patient-related factors (such as advanced age, stroke type, lesion location and extent, and pre-existing comorbidities); biochemical factors (such as excessive excitatory neurotransmitter release, calcium overload, oxidative stress, neuroinflammation, and blood–brain barrier dysfunction); and surgical-related factors (6). In recent years, several novel biomarkers have demonstrated promising applications in the early diagnosis of PSS (7). Traditional assessments of post-stroke status primarily focus on age, degree of neurological deficit, and language/cognitive function. However, these evaluations contain significant subjective elements influenced by individual clinicians, resulting in a lack of unified, objective, standardized predictive value (8). However, most studies on the predictive value of biochemical markers for PSS have focused on sub-healthy populations at risk of stroke, with limited research addressing the prediction of PSS occurrence. Therefore, identifying biomarkers for the early diagnosis and prediction of PSS holds significant importance for guiding clinical treatment and improving prognosis.

At present, with the rapid advancement of artificial intelligence and big data technologies, machine learning is increasingly applied across medical fields (9). As a branch of artificial intelligence, machine learning employs algorithms and models to enable computers to automatically learn from data and make predictions or decisions. In recent years, it has demonstrated promising applications in predicting various brain disorders. For instance, it shows significant potential in diagnosing and forecasting the prognosis of multiple neurological conditions such as stroke and Alzheimer’s disease (9). However, existing machine learning research in stroke has primarily focused on predicting the clinical symptoms of the disease itself, with insufficient attention given to predicting the prognosis of post-stroke sequelae. Additionally, studies commonly suffer from limited sample sizes, single variables, and insufficient population representativeness (44). They also frequently employ single algorithms, lacking structural designs for multi-model integration (10). Research indicates that constructing models through the combination of multiple factors and integrating various machine learning models can significantly improve the accuracy of outcome prediction (11, 47). Therefore, there is an urgent need to develop a model capable of effectively predicting the occurrence of PSS after acute stroke based on a combination of multiple machine learning techniques.

Given the limited existing research on predicting PSS after acute stroke, this study aims to construct a model for predicting the risk of PSS occurrence based on clinical and biochemical indicators of stroke patients, combined with multiple machine learning algorithms (10). By analyzing the relationship between clinical factors (e.g., age, gender) and biochemical factors (e.g., blood glucose, lipid levels) with PSS occurrence, and comprehensively assessing patients’ PSS risk, the accuracy of PSS risk prediction can be further enhanced (7, 12). This approach offers new insights for early identification and intervention of post-stroke sequelae, ultimately improving patient prognosis and quality of life (13). This study holds promise to provide crucial theoretical foundations and practical guidance for advancements in stroke rehabilitation and secondary prevention (46).

## Method

### Patient data sources and preprocessing

This retrospective, single-center study included patients hospitalized for acute stroke in the Department of Neurosurgery at Zhujiang Hospital between 2008 and 2024. Patients were included irrespective of EVT status. Patients were eligible if the index stroke met applicable Chinese or international diagnostic criteria and the required baseline and follow-up records were available. We excluded patients with conditions that precluded meaningful follow-up, pregnancy or lactation, insufficient key baseline information, or no post-admission follow-up. Of 2,773 screened records, 2,095 patients were included. The Ethics Review Committee of Zhujiang Hospital, Southern Medical University approved the study (2024-KY-231-04) and waived written informed consent. Reporting followed TRIPOD+AI guidance (14).

Time zero was the index stroke admission. The primary outcome was time to the first documented persistent post-stroke functional impairment after completion of acute treatment. Persistent impairment was operationally defined as a neurological, motor, language, cognitive/psychological, or activities-of-daily-living impairment attributable to the index stroke and documented in the clinical or follow-up record after acute treatment. The event date was the date of the first qualifying documentation; when multiple qualifying impairments were recorded, the earliest date defined the event. Patients without a documented PSS event were censored at their last available follow-up.

De-identified PSS event indicators and event-or-censoring times were accessed from the clinical database of Zhujiang Hospital through authorized institutional procedures and used for the time-to-event analyses.

Thirty-seven baseline candidate predictors were defined from the index admission, principally using the first eligible laboratory measurement obtained within 24 h. Hypertension and diabetes, when present, were treated as pre-existing comorbidities or vascular risk factors rather than as post-stroke sequelae. Detailed definitions, units, acquisition windows, variable types, and missingness are provided in Supplementary Table S1.

The cohort was split before preprocessing and model development. Variables with more than 15% missingness were excluded. For retained variables, random-forest imputation was estimated in the training data; derived ratios were calculated after component imputation. Transformation and standardization parameters estimated in the training data were applied unchanged to the internal test data. Feature selection and model tuning were conducted in the training cohort, and the internal test cohort was reserved for final evaluation (15).

### Variable selection and model construction for the post-stroke sequelae risk score (PSSRS)

The dataset was randomly divided by stratified allocation into a training set (70%; *n* = 1,467) and a locked internal test set (30%; *n* = 628). All feature selection and model tuning were conducted in the training set. Univariable Cox screening retained the 13 variables displayed in Figure 1B at the reported *p* ≤ 0.05 boundary. Multivariable Cox screening retained 16 variables at *p* < 0.20. LASSO with 10-fold cross-validation retained 10 non-zero coefficients, and RSF variable-importance ranking retained the top 50%, corresponding to 18 variables (16–19). Eight variables selected by all four methods were retained: HCY, CK-MB, LDH, GGT, HDL-C, Glu (venous), DBIL, and CK-MB/CK. The intersection was prespecified as a conservative stability filter across complementary methods. Method-specific selections are reported in Supplementary Table S2 (19).

**Figure 1:**
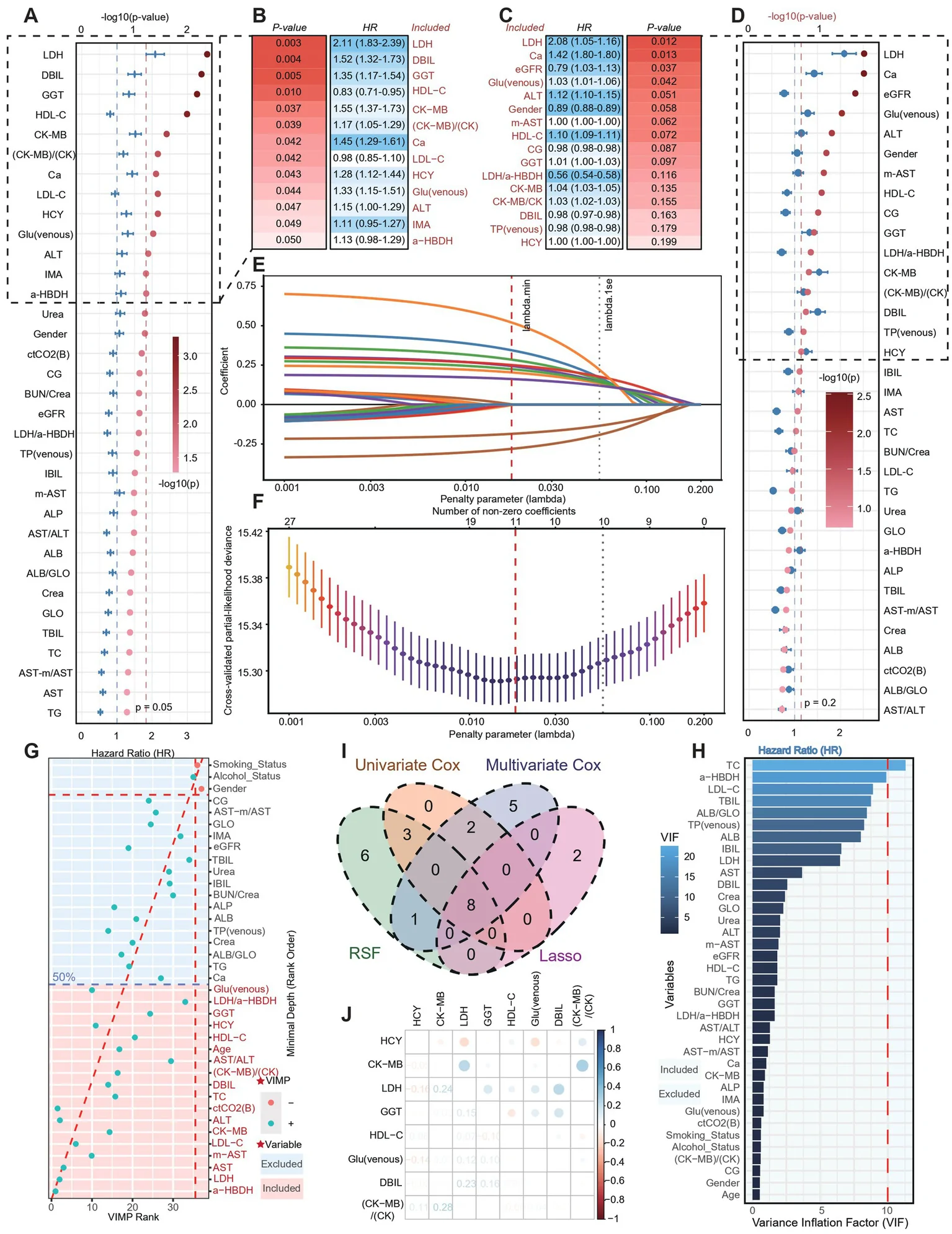
Four-method screening and correlation assessment of candidate predictors for PSSRS. **(A–D)** Univariable and multivariable Cox regression results. **(A)** Univariate Cox analysis results (top-ranked variables), showing *p*-values and hazard ratios (HR, with 95% confidence intervals) for most candidate variables in univariate regression. **(B,C)** Summary plots of variables included in univariate and multivariate regression. Red denotes variables retained by the stated screening criterion. Univariable inclusion criterion: the 13 variables displayed at the reported *p* ≤ 0.05 boundary; multivariable screening criterion: *p* < 0.20. **(D)** Multivariable Cox regression results (top-ranked variables), displaying *p*-values and hazard ratios (with 95% confidence intervals) for most candidate variables in the multivariable analysis. **(E,F)** LASSO regression analysis. **(E)** Feature shrinkage path diagram; **(F)** Cross-validation plot identifying 10 non-zero coefficient variables. **(G)** Random survival forest (RSF) variable-importance screen; variables above the prespecified rank threshold were retained. **(H)** Variance inflation factor (VIF) assessment of the final predictors; the red reference line marks VIF = 10. **(I)** Venn diagram illustrating the intersection of four methods: univariate Cox, multivariate Cox, LASSO, and RSF, ultimately identifying eight stable predictors. **(J)** Spearman correlation matrix for the eight shared predictors; the prespecified absolute-correlation criterion was |r| < 0.60.

We compared 101 prespecified survival-model pipelines. Model ranking was based on training-set resampling, with cross-validated Harrell C-index as the primary criterion. Time-dependent AUROC, AUPRC, Brier score, and calibration were reserved for assessment of the final model. Harrell C-index was computed as a global rank-discrimination measure over observed follow-up within each cohort. Training and internal-test C-indices are reported separately. Algorithm, feature, hyperparameter, and threshold selection were completed in the training set; the StepCox (forward) + RSF pipeline was then locked and evaluated once in the internal test set. The complete pipeline list and hyperparameter search space are reported in Supplementary Tables S3–S5. For the selected RSF component, the reported configuration used 1,000 trees, node size 5, mtry 3, 10 random split points, sampling fraction 0.632, and the log-rank split rule; all choices were made inside training-set resampling.

### Model evaluation and interpretation of PSSRS

Discrimination was evaluated using Harrell C-index and time-dependent AUROC at 1, 3, and 5 years. AUPRC was added because it is informative when event prevalence is imbalanced, and prediction error was quantified using censoring-aware Brier scores. Test-set uncertainty was estimated with 1,000 patient-level bootstrap resamples. Calibration was assessed graphically and with calibration intercept, calibration slope, expected-to-observed (E/O) ratio, and the Greenwood-Nam-D’Agostino goodness-of-fit test. Decision curve analysis compared the model with treat-all and treat-none strategies across clinically relevant threshold probabilities. Any binary decision threshold was selected from training predictions and applied unchanged to the test set (20–23).

Five-fold and 10-fold cross-validation were performed within the training set as sensitivity analyses. Preprocessing and feature-selection steps were refitted within each resample, and fold-level performance was summarized by the mean and standard deviation (24).

SHAP (SHapley Additive exPlanations) analysis was performed to characterize predictor contributions to the fitted random survival forest model (42). StepCox (forward) provided upstream feature screening, and the retained variables were entered into the RSF for model fitting and prediction. SHAP values therefore describe contributions to the RSF prediction output. The training cohort served as the background/reference dataset for the SHAP explainer, and SHAP values were calculated for patients in the internal test cohort. Global feature importance was summarized using mean absolute SHAP values, and the beeswarm plot displayed the direction and distribution of individual contributions. Decision and force plots illustrated local prediction patterns; two internal-test cases illustrated high- and low-risk predictions.

### Construction of the simplified PSSRS nomogram and shiny application

To provide a lower-burden clinical presentation, we developed a simplified Cox nomogram using LDH, CK-MB, and DBIL, the three variables shared by the leading RSF-importance and mean-absolute-SHAP rankings (25–27). HCY remained a predictor in the full PSSRS; the simplified nomogram used the three variables in the shared top-ranked subset. The simplified model was fitted in the training set and evaluated separately in the internal test set. The simplified nomogram provided a more accessible representation of the model using fewer predictors. The simplified nomogram was deployed as an interactive Shiny application to provide individualized 1-, 3-, and 5-year PSS-free probability estimates (28). The application is available at https://stroke.shinyapps.io/DynNom_Sequelae_of_Stroke/.

### Statistical analysis

All statistical analyses were performed using R 4.3.1, with Python used for SHAP visualization. Continuous variables are reported as median (interquartile range) and categorical variables as count (percentage). Training and test cohorts were compared descriptively using the Wilcoxon rank-sum test for continuous variables and the chi-square or Fisher exact test for categorical variables, as appropriate.

PSS-free probability was estimated using the Kaplan–Meier method, and cumulative event probability was displayed as 1 minus the Kaplan–Meier estimate. The risk-group cutoff was determined in the training set and applied unchanged to the internal test set. Group differences were assessed using the log-rank test (29). Discrimination, prediction error, calibration, and clinical utility were evaluated using the metrics described above. All confidence intervals were two-sided 95% intervals, and *p* < 0.05 was used for inferential tests. Software versions, the random seed, and model settings are reported in the Supplementary methods and Supplementary Table S5.

## Result

### Basic clinical information of stroke patients

Among 2,095 included patients, 1,050 had a documented PSS event and 1,045 had no documented PSS event during observed follow-up and were right-censored. Baseline characteristics stratified by observed outcome are presented in Table 1. The descriptive comparison of the training and internal test cohorts is reported separately in Supplementary Table S12. Outcome counts and follow-up summaries, including an overall median follow-up of 1,175 days (IQR, 602–1,626 days), are provided in Supplementary Table S13. The full candidate-variable definitions and preprocessing rules are provided in Supplementary Table S1, and the study workflow is shown in Figure 2.

**Table 1:** Baseline characteristics of patients included in the PSSRS cohort, stratified by documented PSS outcome.

Characteristic	Overall cohort (*N* = 2,095)	Documented PSS event (*n* = 1,050)	No documented PSS event/censored (*n* = 1,045)	*p* value
Stroke characteristics				
Stroke subtype				
Ischemic stroke	1,950 (93.1%)	970 (92.4%)	980 (93.8%)	0.214
Hemorrhagic stroke	145 (6.9%)	80 (7.6%)	65 (6.2%)	
Previous stroke history				
No	1,720 (82.1%)	830 (79.0%)	890 (85.2%)	<0.001
Yes	375 (17.9%)	220 (21.0%)	155 (14.8%)	
Time from symptom onset to admission, hours	12.0 (5.0, 28.0)	13.0 (6.0, 30.0)	11.0 (5.0, 26.0)	0.072
Clinical severity at admission				
NIHSS score at admission	5 (2, 9)	6 (3, 11)	4 (2, 7)	<0.001
Comorbidities				
Hypertension	1,350 (64.4%)	710 (67.6%)	640 (61.2%)	0.003
Diabetes mellitus	510 (24.3%)	280 (26.7%)	230 (22.0%)	0.012
Atrial fibrillation	180 (8.6%)	105 (10.0%)	75 (7.2%)	0.018
Coronary artery disease	220 (10.5%)	125 (11.9%)	95 (9.1%)	0.034
Smoking status				
No	1,426 (68.1%)	690 (65.7%)	736 (70.4%)	0.021
Yes	669 (31.9%)	360 (34.3%)	309 (29.6%)	
TC (mmol/L)	4.38 (3.63, 5.18)	4.35 (3.61, 5.18)	4.41 (3.65, 5.19)	0.235
a-HBDH (U/L)	180 (150, 220)	193 (159, 238)	170 (145, 203)	<0.001
LDL-C (mmol/L)	2.56 (1.93, 3.26)	2.65 (2.00, 3.32)	2.48 (1.88, 3.20)	0.004
TBIL (μmol/L)	12.4 (9.5, 16.2)	12.7 (9.7, 16.6)	12.1 (9.3, 16.0)	0.062
ALB/GLO (ratio)	1.33 (1.12, 1.58)	1.32 (1.11, 1.57)	1.34 (1.13, 1.59)	0.356
TP (venous) (g/L)	67.0 (62.0, 71.5)	67.2 (62.2, 71.8)	66.8 (61.8, 71.2)	0.091
ALB (g/L)	41.2 (37.7, 44.7)	41.0 (37.5, 44.5)	41.4 (37.9, 44.9)	0.151
IBIL (μmol/L)	8.3 (5.9, 11.1)	8.5 (6.0, 11.3)	8.2 (5.8, 10.9)	0.122
LDH (U/L)	185 (164, 206)	201 (174, 233)	170 (151, 191)	<0.001
AST (U/L)	20 (16, 27)	21 (16, 28)	20 (16, 26)	0.032
DBIL (μmol/L)	4.06 (3.00, 5.00)	4.47 (3.30, 5.40)	3.73 (2.80, 4.60)	<0.001
Crea (μmol/L)	78 (66, 94)	80 (67, 96)	76 (65, 92)	0.008
GLO (g/L)	30.8 (27.5, 34.6)	30.9 (27.6, 34.8)	30.7 (27.4, 34.4)	0.465
Urea (mmol/L)	5.5 (4.3, 7.0)	5.7 (4.4, 7.2)	5.3 (4.2, 6.8)	0.009
ALT (U/L)	19 (14, 28)	20 (15, 29)	18 (14, 27)	0.041
m-AST (U/L)	10.0 (7.0, 14.0)	10.2 (7.1, 14.3)	9.9 (6.9, 13.8)	0.071
eGFR (mL/min/1.73 m^2^)	90.2 (75.0, 103.4)	87.5 (72.3, 101.2)	93.0 (77.5, 105.1)	0.004
HDL-C (mmol/L)	1.05 (0.91, 1.23)	1.00 (0.87, 1.18)	1.10 (0.95, 1.27)	<0.001
TG (mmol/L)	1.47 (1.05, 2.08)	1.50 (1.07, 2.11)	1.44 (1.04, 2.05)	0.097
BUN/Crea (ratio)	0.070 (0.055, 0.090)	0.071 (0.055, 0.091)	0.069 (0.054, 0.089)	0.188
GGT (U/L)	30 (20, 41)	34 (22, 46)	27 (19, 37)	<0.001
LDH/a-HBDH (ratio)	1.03 (0.86, 1.22)	1.04 (0.87, 1.23)	1.02 (0.85, 1.21)	0.097
AST/ALT (ratio)	1.12 (0.82, 1.55)	1.13 (0.82, 1.57)	1.11 (0.81, 1.53)	0.441
HCY (μmol/L)	12.0 (9.8, 13.7)	12.7 (10.3, 14.4)	11.4 (9.4, 13.1)	<0.001
AST-m/AST (ratio)	0.50 (0.35, 0.68)	0.50 (0.35, 0.68)	0.50 (0.35, 0.68)	0.622
Ca (mmol/L)	2.28 (2.18, 2.37)	2.27 (2.17, 2.36)	2.29 (2.19, 2.38)	0.011
CK-MB (U/L)	14 (10, 17)	15 (11, 18)	12 (9, 16)	<0.001
ALP (U/L)	79 (64, 99)	80 (65, 100)	78 (63, 98)	0.195
IMA (U/mL)	0.45 (0.39, 0.52)	0.47 (0.40, 0.54)	0.44 (0.38, 0.51)	0.013
Glu (venous) (mmol/L)	5.52 (4.80, 6.83)	5.87 (5.02, 7.21)	5.19 (4.61, 6.39)	<0.001
ctCO2(B) (mmol/L)	23.4 (21.1, 25.7)	23.1 (20.9, 25.4)	23.7 (21.4, 26.0)	0.023
Alcohol status	No: 1,642 (78.4%); Yes: 453 (21.6%)	No: 815 (77.6%); Yes: 235 (22.4%)	No: 827 (79.1%); Yes: 218 (20.9%)	0.425
CK-MB/CK (%)	21 (14, 25)	23 (16, 28)	19 (13, 23)	<0.001
CG (μg/mL)	1.00 (0.85, 1.16)	1.02 (0.86, 1.18)	0.98 (0.84, 1.14)	0.084
Sex	Female: 709 (33.8%); Male: 1,386 (66.2%)	Female: 350 (33.3%); Male: 700 (66.7%)	Female: 359 (34.4%); Male: 686 (65.6%)	0.592
Age (years)	63 (55, 71)	65 (57, 73)	62 (54, 70)	0.018

Data are median (IQR) or n (%). The overall cohort comprised 1,050 documented PSS events and 1,045 right-censored observations; median follow-up was 1,175 days (IQR, 602–1,626 days; Supplementary Table S13). *p* values compare documented-event and right-censored groups.

Data are presented for the overall cohort, patients with a documented PSS event, and patients with no documented PSS event during observed follow-up who were right-censored. Continuous variables are summarized as median (interquartile range) and categorical variables as count (percentage). *p* values describe unadjusted between-group comparisons. Definitions and missingness for the candidate variables are provided in Supplementary Table S1.

**Figure 2:**
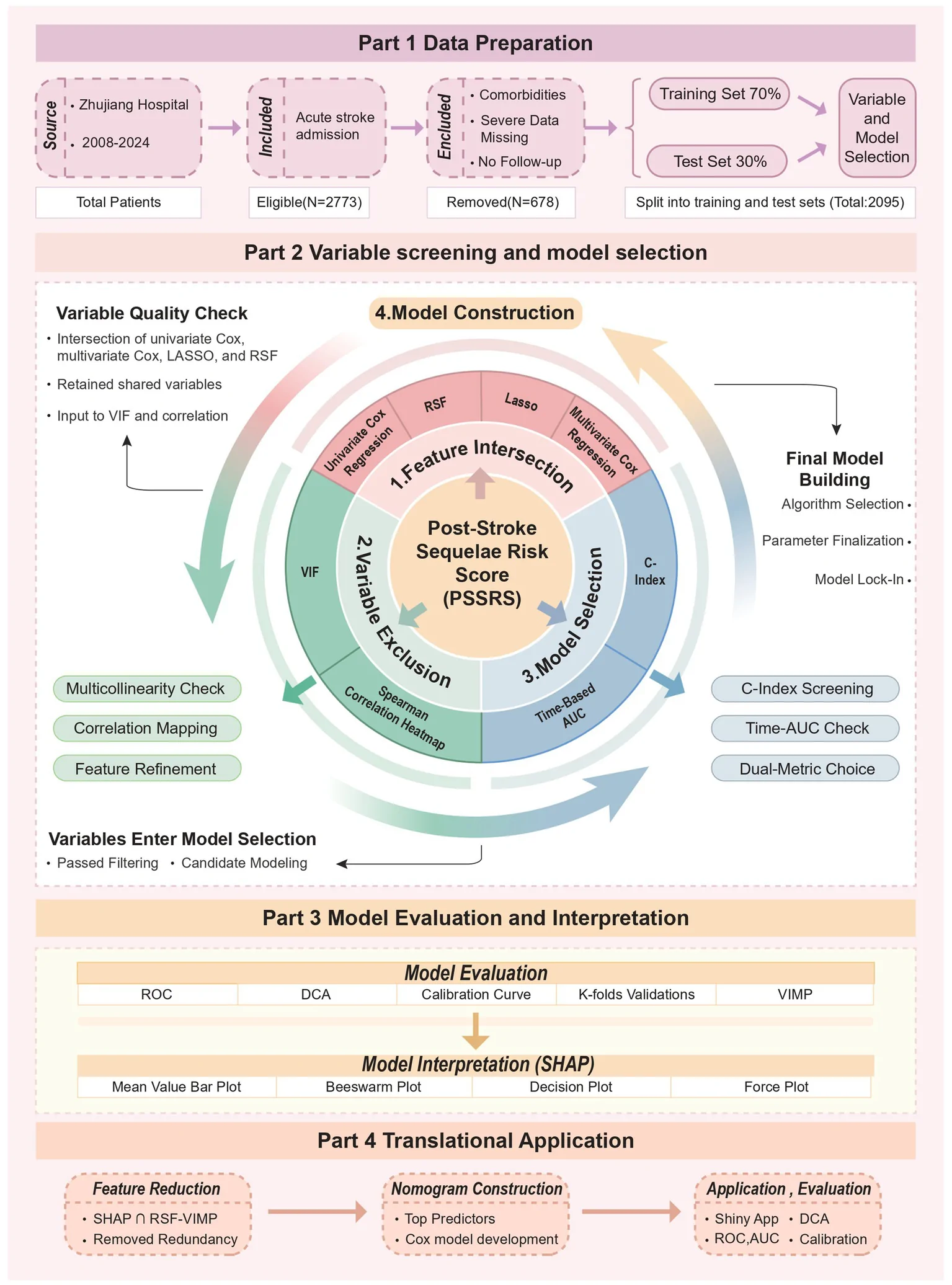
Study design and development of the Post-Stroke Sequelae Risk Score (PSSRS). The workflow shows cohort screening (2,773 screened; 2,095 included), the prespecified 7:3 training/internal-test split, training-set preprocessing and four-method feature selection, and candidate-pipeline comparison and locking. The final StepCox (forward) + RSF model used eight predictors and was evaluated once in the internal test cohort.

### Model construction and performance comparison of PSSRS

The four training-set feature-selection procedures retained 13 variables by univariate Cox analysis, 16 by multivariable Cox analysis, 10 by LASSO, and 18 by RSF importance. Their intersection comprised HCY, CK-MB, LDH, GGT, HDL-C, Glu (venous), DBIL, and CK-MB/CK (Figure 1). VIF and Spearman correlation analyses did not identify a prespecified threshold violation among the final eight variables; nevertheless, the related CK-MB and CK-MB/CK terms were retained with caution. Sensitivity comparison showed that the eight-variable intersection model had a internal-test C-index of 0.850 and 1-, 3-, and 5-year AUROCs of 0.800, 0.830, and 0.820, respectively; corresponding values were 0.842 and 0.791, 0.818, and 0.808 for the 27-variable union model and 0.831 and 0.781, 0.806, and 0.795 for the 37-variable full-candidate model (Supplementary Table S14).

Across the 101 prespecified candidate pipelines, StepCox (forward) + RSF had the highest training-set resampling performance and was locked before internal-test evaluation (Figures 3A). The final model had a C-index of 0.85 in the internal test cohort; its time-dependent AUROCs are reported in the performance-validation results (Figures 3E–H). Cumulative PSS-event curves based on the training-derived risk cutoff separated the predicted high- and low-risk groups in the training cohort (733 vs. 734) and internal test cohort (391 vs. 237) (log-rank *p* < 0.0001 and *p* = 0.00051, respectively). The overall cohort included 1,124 high-risk and 971 low-risk patients at day 0 (Figures 3B–D).

**Figure 3:**
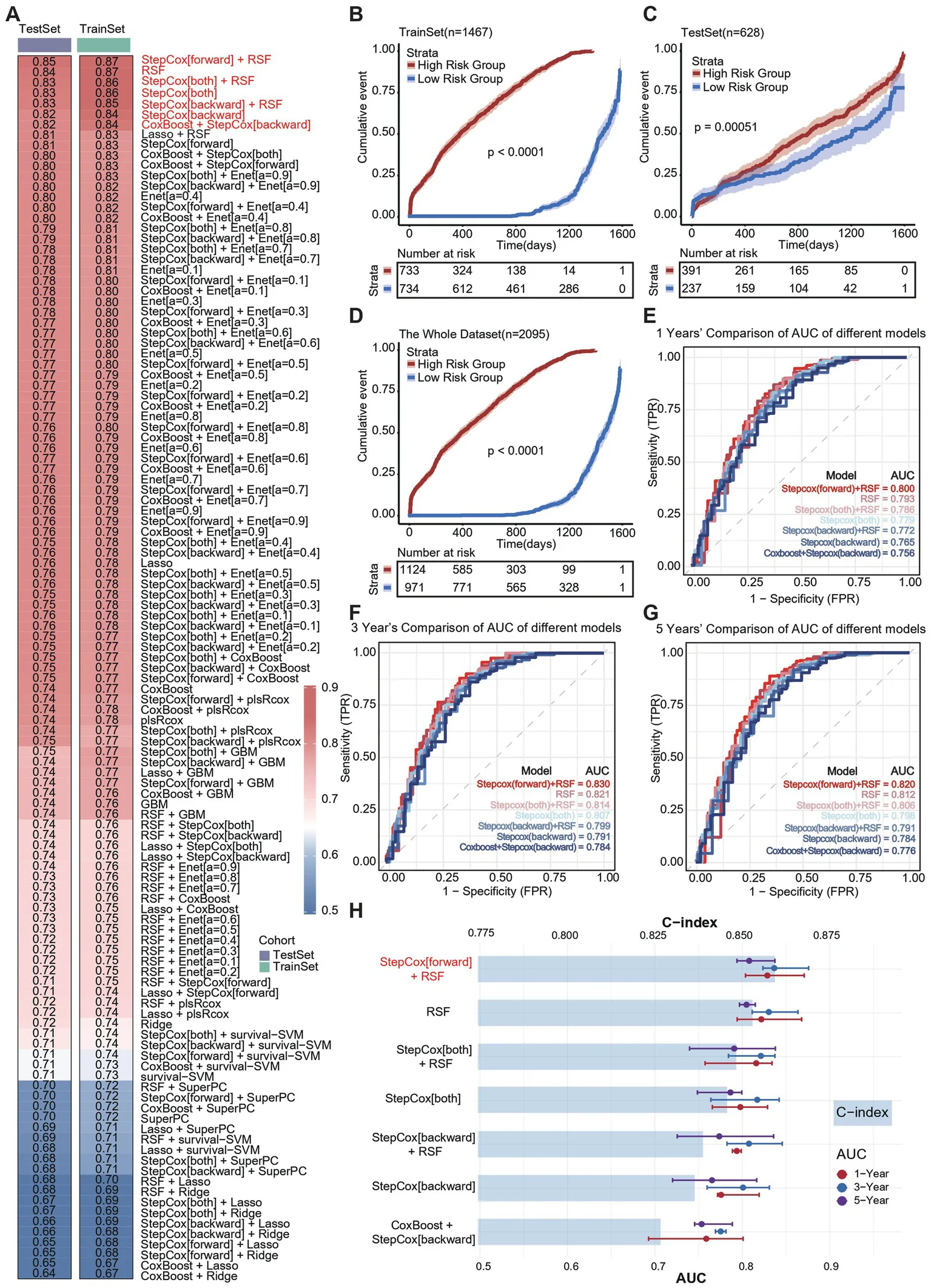
Candidate-model comparison and risk-group stratification of PSSRS. **(A)** C-index heatmap for the 101 candidate pipelines. Training-set and internal-test-set C-index columns are displayed. **(B–D)** Cumulative PSS-event curves for predicted-risk groups in the training set (high *n* = 733; low *n* = 734), internal test set (high *n* = 391; low *n* = 237), and overall-cohort panel (high *n* = 1,124; low *n* = 971). The cutoff was derived in the training cohort and applied unchanged to the internal test cohort; corresponding numbers at risk are reported in Supplementary Table S8. **(E–G)** Time-dependent ROC curves at 1, 3, and 5 years comparing the six displayed models in the internal test cohort. **(H)** C-index and 1-, 3-, and 5-year AUROC summary for the six displayed models.

### Predictive performance evaluation of PSSRS

Training-set AUROCs were 0.850, 0.880, and 0.870 (Figure 4A). In the internal test set, AUROCs were 0.800 (95% CI, 0.760–0.840), 0.830 (0.790–0.870), and 0.820 (0.780–0.860), respectively (Figure 4B). Corresponding test-set AUPRCs were 0.748, 0.772, and 0.761, and Brier scores were 0.140, 0.170, and 0.180 (Supplementary Table S6).

**Figure 4:**
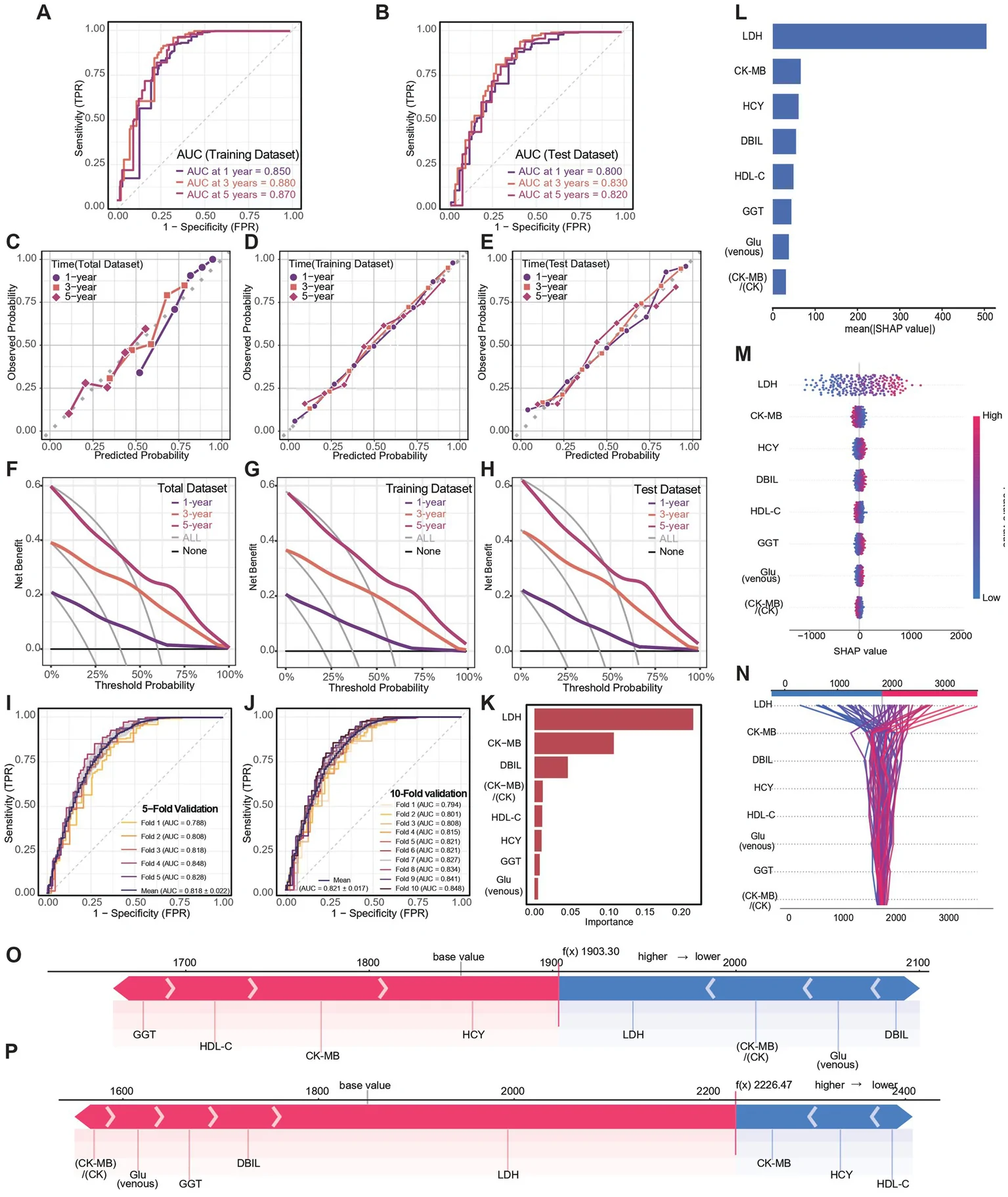
Internal performance assessment and interpretability analysis of the PSSRS. **(A)** Time-dependent ROC curves for the training cohort. Training-set AUROCs were 0.850, 0.880, and 0.870 at 1, 3, and 5 years, respectively. **(B)** Time-dependent ROC curves for the internal test cohort; internal-test AUROCs were 0.800, 0.830, and 0.820 at 1, 3, and 5 years, respectively. **(C–E)** Calibration plots for the overall, training, and internal-test cohorts, respectively; intercept, slope, E/O ratio, and goodness-of-fit results are reported in Supplementary Table S7. **(F–H)** Decision-curve analyses for the overall, training, and internal-test cohorts, respectively, compared PSSRS with treat-all and treat-none strategies across threshold probabilities. **(I,J)** Fivefold and tenfold training-set cross-validation, with mean held-out AUROC of 0.818 (SD 0.022) and 0.821 (SD 0.017), respectively. **(K)** RSF variable-importance ranking for all eight predictors in the locked PSSRS. **(L)** Global SHAP feature importance summarized using mean absolute SHA*p* values calculated in the internal test cohort. StepCox (forward) was used only for upstream feature screening, and all displayed SHA*p* values explain the prediction output of the fitted RSF model. **(M)** SHAP beeswarm plot showing the direction and distribution of individual feature contributions to RSF predictions. **(N)** SHAP decision plot illustrating the cumulative contribution of predictors to the RSF output. **(O,P)** Local SHAP force plots for one illustrative high-risk patient and one illustrative low-risk patient from the internal test cohort. Red and blue segments indicate contributions that increase and decrease the RSF prediction output, respectively. Local SHAP force plots were provided to illustrate individual prediction patterns.

In the internal test set, calibration intercepts at 1, 3, and 5 years were 0.02, 0.05, and 0.08; calibration slopes were 0.96, 0.93, and 0.90; and E/O ratios were 1.02, 1.04, and 1.07, respectively (Figures 4C–E; Supplementary Table S7). Decision curves showed higher net benefit than the treat-all and treat-none strategies across portions of the displayed clinically relevant threshold range (Figures 4F–H). Mean five-fold and 10-fold training-set cross-validation performance was 0.818 (SD 0.022) and 0.821 (SD 0.017), respectively (Figures 4I,J), supporting similar performance across the two resampling schemes.

### Exploration of key biomarkers in PSSRS

RSF variable importance and Python-based SHAP analyses provided complementary descriptions of predictor contribution (Figures 4K–M). The mean absolute SHAP plot summarized global importance, whereas the beeswarm plot displayed the direction and heterogeneity of individual contributions. LDH, HCY, CK-MB, and DBIL were among the leading SHAP features, while LDH, CK-MB, and DBIL were also prominent in the RSF ranking. The decision plot illustrated cumulative feature contributions to model output (Figure 4N). Force plots for one high-risk and one low-risk internal-test patient illustrate individual prediction patterns (Figures 4O,P).

### Contribution of PSSRS to clinical support

The simplified nomogram included LDH, CK-MB, and DBIL (Figures 5A–C). In the internal test cohort, its 1-, 3-, and 5-year AUROCs were 0.750, 0.780, and 0.770, respectively (Figure 5D), an absolute decrease of 0.050 at each horizon relative to the full PSSRS. Paired patient-level bootstrap comparisons (1,000 resamples) yielded 95% confidence intervals that included zero at all horizons (*p* = 0.18, 0.16, and 0.11, respectively; Supplementary Table S15). Calibration and decision curves are presented as descriptive internal-validation results (Figures 5E,F). The simplified nomogram is also available as an interactive Shiny application for individualized 1-, 3-, and 5-year PSS-free probability estimation at https://stroke.shinyapps.io/DynNom_Sequelae_of_Stroke/.

**Figure 5:**
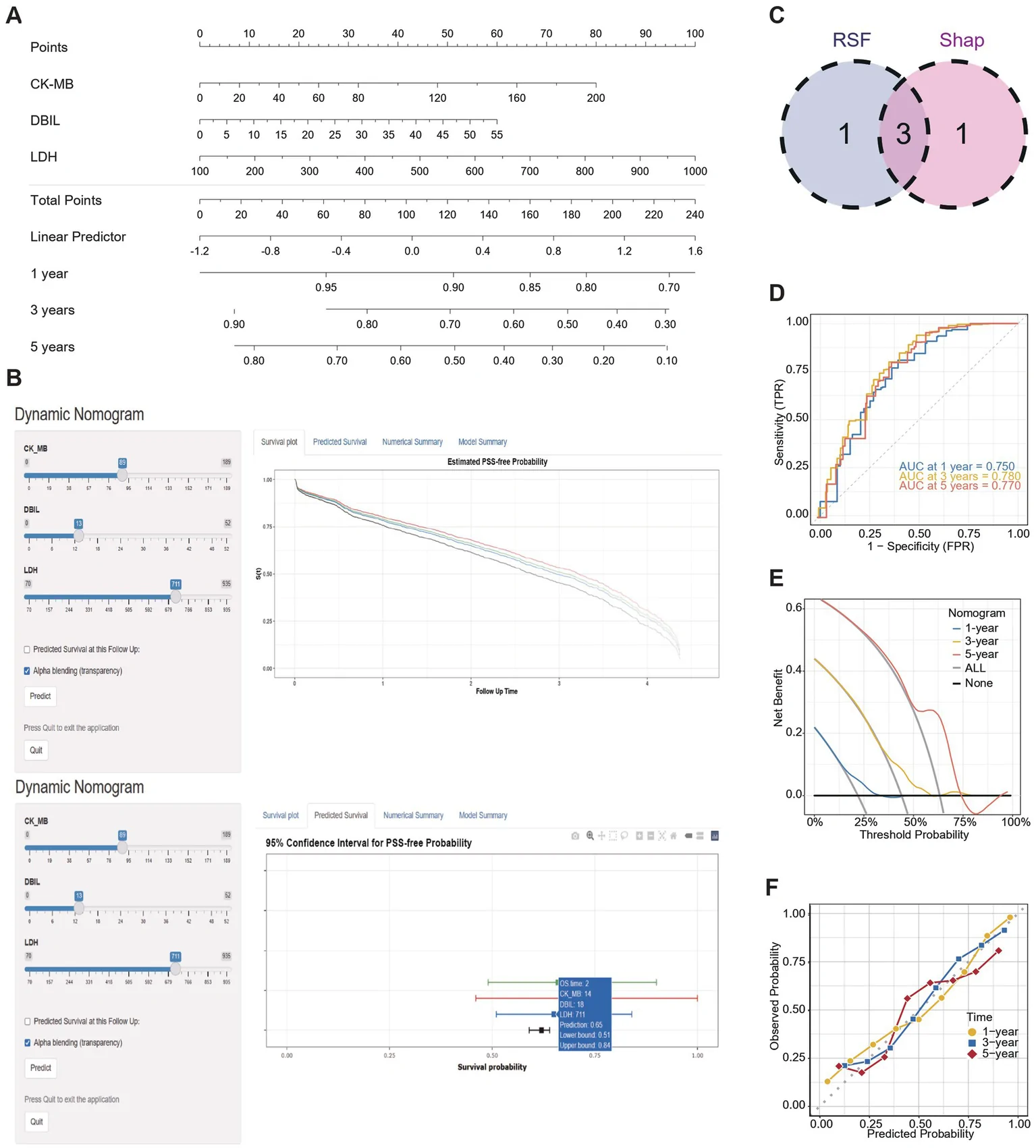
Simplified Cox nomogram and internal-test evaluation. **(A)** Simplified nomogram based on LDH, CK-MB, and DBIL for estimated PSS-free probability at 1, 3, and 5 years. **(B)** Interactive Shiny dynamic-nomogram interface for individualized 1-, 3-, and 5-year PSS-free probability estimates and confidence intervals. The application is available at https://stroke.shinyapps.io/DynNom_Sequelae_of_Stroke/. In the package-generated interface, “survival” refers to PSS-free probability. **(C)** Overlap between the leading RSF-importance and mean-absolute-SHAP features; the shared predictors LDH, CK-MB, and DBIL define the simplified predictor set. **(D)** Internal-test ROC curves for the simplified nomogram, with AUROCs of 0.750, 0.780, and 0.770 at 1, 3, and 5 years, respectively. **(E)** Internal-test decision curve analysis for the simplified nomogram. **(F)** Internal-test calibration curves. The simplified nomogram provided a more accessible representation of the model using fewer predictors.

### Discussion

This study developed a time-to-event model for PSS among patients hospitalized with acute stroke, including but not limited to those who underwent EVT. Among 101 candidate pipelines, StepCox (forward) + RSF showed the numerically highest training-resampling performance and was selected before internal-test evaluation (30, 31). The internal-test AUROCs of 0.800, 0.830, and 0.820 at 1, 3, and 5 years, together with a C-index of 0.85, indicate moderate-to-good discrimination. The modest difference from the training C-index of 0.87 reflects expected optimism in model development. These findings highlight the potential value of routinely collected biochemical markers for post-stroke risk stratification and provide a basis for future external validation (32).

In constructing the PSSRS, we identified LDH, CK-MB, DBIL, CK-MB/CK, HCY, GGT, HDL-C, and admission Glu (venous) as the eight retained predictors associated with PSS risk. These findings align with previous studies indicating that hyperglycemia during admission or peri-treatment periods is significantly associated with short- and long-term adverse outcomes in acute ischemic stroke, and similarly predicts poorer functional recovery in patients undergoing mechanical thrombectomy (33). Elevated LDH correlates with higher NIHSS/mRS scores and poor prognosis, suggesting tissue injury and metabolic stress levels play a crucial role in outcomes (34, 48). As a surrogate marker for oxidative stress and glutathione metabolism, increased GGT is associated with heightened stroke risk, supporting the potential role of the oxidative-inflammatory pathway in PSS development (35). Regarding bilirubin, recent reviews and meta-analyses indicate higher bilirubin levels correlate positively with stroke severity, hemorrhagic transformation, and adverse outcomes. A complex nonlinear relationship may exist between HDL-C and stroke prognosis (36). Studies indicate a U-shaped association between HDL-C levels and functional outcomes in ischemic stroke patients, where both excessively low and high HDL-C levels may correlate with poorer neurological recovery. This aligns with our observed trend, suggesting HDL-C may exert bidirectional regulatory effects on stroke prognosis (37). Regarding homocysteine (Hcy), although no direct evidence of causality between Hcy and stroke exists in clinical studies, research indicates Hcy may impair vascular endothelial function and reduce nitric oxide (NO) production, leading to vascular stiffening and impaired blood perfusion. These factors may contribute to poor stroke outcomes and promote the development of post-stroke sequelae (38). Regarding myocardial injury markers, although systematic research linking these markers to stroke prognosis remains limited, our findings indicate that elevated total CK and CK-MB levels correlate closely with stroke recurrence, mortality, and poor functional recovery (39, 40). Concurrently, the (CK-MB)/CK ratio demonstrates unique potential in reflecting activation of the “neurogenic myocardial injury-brain-heart axis,” offering significant insights into elucidating post-stroke brain-heart interaction mechanisms and identifying novel prognostic biomarkers (40). By integrating multidimensional features including metabolic stress (blood glucose, LDH), oxidative/antioxidant balance (GGT, bilirubin, HDL-C), amino acid metabolism (Hcy), and myocardial injury signals (CK-MB and its ratio), PSSRS enables a more comprehensive characterization of the PSS risk spectrum, providing a basis for stratified management and personalized interventions. Future multicenter external validation will be important to assess its transferability, and causal-inference and mechanistic studies may further elucidate the contribution of these pathways to PSS.

Several limitations should be considered. This single-center retrospective study was internally validated and requires geographic and temporal validation. The 16-year recruitment period may encompass changes in assays, treatment pathways, and follow-up practice that a random split cannot fully assess. Outcome ascertainment from retrospective records may be heterogeneous. Selection among 101 candidate pipelines may introduce selection optimism despite training-set resampling. The intersection-based strategy and related CK-MB and CK-MB/CK predictors warrant further evaluation; sensitivity analyses using alternative feature sets and removal of either related term are reported in Supplementary Tables S9, S14. The simplified nomogram showed lower discrimination than the full PSSRS, and SHAP analyses characterize model contributions. Further validation in independent cohorts is needed to confirm model robustness. Prospective, multicenter validation with standardized PSS adjudication and model updating when needed will be important to evaluate generalizability and clinical applicability.

## Conclusion

Among the evaluated pipelines, StepCox (forward) + RSF showed the strongest prespecified training-resampling performance and moderate-to-good internal-test discrimination for PSS. The retained predictors were LDH, CK-MB, DBIL, CK-MB/CK, HCY, GGT, HDL-C, and Glu (venous). PSSRS provides an internally validated framework for individualized PSS risk estimation, with a simplified dynamic nomogram offering a lower-burden representation. Independent temporal and multicenter validation will be important to evaluate its generalizability and clinical applicability.

## Data Availability

The raw data supporting the conclusions of this article will be made available by the authors, without undue reservation.
