# Supplementary material for: Post-stroke sequelae risk score (PSSRS): a 16-year retrospective study developing an internally validated multi-algorithm model for post-stroke sequelae
Source: Front Neurol. 2026 Oct 15;17:1833445. doi: 10.3389/fneur.2026.1833445 (PMC13716776; doi:10.3389/fneur.2026.1833445)
Supplement: Supplementary file 1 [file Supplementary_file_1.pdf]

1. Supplementary Material

Title: PSSRS: A 16-Year Retrospective Study Developing an Internally Validated Multi-Algorithm Model for Post-Stroke Sequelae

Correspondence:

Dr. Xifeng Li
Email: nflxf@126.com

Dr. Yanchao Liu
Email: liuyanchao_zj@126.com

Dr. Chuanzhi Duan
Email: doctor_duanzj@163.com

2. Supplementary Methods

Post-Stroke Sequelae Risk Score: Model Development and Internal Validation

Design: Retrospective, single-center, time-to-event prediction-model study
Cohort: 2,095 patients hospitalized with acute stroke in the Department of Neurosurgery at Zhujiang Hospital between 2008 and 2024
Data split: Training cohort, n = 1,467; internal test cohort, n = 628; random seed = 712
Final pipeline: Forward stepwise Cox regression followed by random survival forest
Prediction horizons: 1, 3, and 5 years after the index stroke admission

2.1. Study Design, Setting, and Reporting Framework

This retrospective, single-center cohort study developed and internally validated a time-to-event prediction model for persistent post-stroke sequelae (PSS). The study population comprised patients hospitalized with acute stroke in the Department of Neurosurgery at Zhujiang Hospital between 2008 and 2024, irrespective of endovascular thrombectomy status.

The study was reported in accordance with TRIPOD+AI recommendations, including the description of data sources, participant selection, outcome definition, predictor assessment, model development, internal validation, model performance, and limitations.

2.2. Participants and Cohort Derivation

Patients were eligible if the index stroke met applicable Chinese or international diagnostic criteria and sufficient baseline and follow-up data were available for predictor and outcome assessment. Exclusion criteria included conditions that prevented meaningful follow-up, pregnancy or lactation, insufficient key baseline information, and absence of post-admission follow-up.

Of 2,773 records screened, 2,095 patients were included in the final cohort. The study was approved by the Ethics Review Committee of Zhujiang Hospital, Southern Medical University (No. 2024-KY-231-04), which waived the requirement for written informed consent because of the retrospective study design.

2.3. Outcome, Time Origin, and Censoring

Time zero was defined as the date of the index admission for acute stroke. The primary endpoint was time to the first documented persistent functional impairment attributable to the index stroke after completion of acute treatment.

Qualifying impairments included persistent neurological, motor, language, cognitive, psychological, or activities-of-daily-living dysfunction documented in the medical or follow-up records. For patients with more than one qualifying impairment, the earliest qualifying documented event date was used in the primary analysis.

Patients without a documented PSS event were right-censored at the date of their last available follow-up. Model performance was assessed at 1, 3, and 5 years after the index admission.

Patient-level PSS event indicators and corresponding event-or-censoring times were retained in the analytical dataset for the time-to-event analyses. Supplementary Table S8 and Supplementary Figure S3 summarize horizon-specific numbers at risk for the risk groups, and Supplementary Table S13 reports event, censoring, and follow-up statistics for the overall, training, and internal test cohorts. PSS-free probability was estimated using the Kaplan–Meier method, and cumulative event probability was calculated as 1 minus the Kaplan–Meier estimate.

2.3.1 Outcome Data and Analytical Dataset

De-identified patient-level outcome data were accessed through authorized institutional procedures. The analytical dataset included the PSS event indicator, date of first documented PSS event where applicable, and time from index admission to the event or last available follow-up for each included patient.

The analytical dataset was used throughout the analytical workflow, including cohort allocation, model development, internal validation, and generation of the reported tables and figures.

Event status and event-or-censoring time were used as outcome variables in the time-to-event analyses.

2.4. Candidate Predictors and Acquisition Window

Candidate predictors were prespecified clinical and biochemical variables available at the index admission. Laboratory predictors were defined primarily as the first eligible measurement obtained within 24 hours after admission.

The candidate set comprised 37 prespecified demographic, behavioral, clinical, and biochemical variables available at the index admission. It included demographic and behavioral characteristics, liver and kidney function indicators, protein-related measures, lipid and metabolic variables, and cardiac or tissue-injury markers. Supplementary Table S1 provides the complete variable dictionary, including definitions, units, data types, acquisition or derivation windows, preprocessing rules, and inclusion in the final eight-predictor set.

Derived ratios were calculated after the component variables had been checked and imputed. These included the albumin-to-globulin ratio, blood urea nitrogen-to-creatinine ratio, LDH-to-alpha-HBDH ratio, AST-to-ALT ratio, non-mitochondrial AST-to-total AST ratio, and CK-MB-to-total CK ratio.

2.5. Data Partitioning and Model Development

Before model development, the 2,095 patients were randomly divided in a stratified 7:3 ratio into a training cohort (n = 1,467) and a locked internal test cohort (n = 628); the random seed was 712. The analytical dataset contained the predictor variables, PSS event indicators, and event-or-censoring times required for the time-to-event analyses.

Imputation, transformation, standardization, feature selection, model fitting, hyperparameter tuning, and threshold selection were performed in the training cohort and repeated within each resampling fold. Event status and event-or-censoring time were treated as outcome information during model development.

The internal test cohort was reserved for final evaluation after the analytical pipeline and risk-group cutoff were determined in the training cohort.

2.6. Missing Data, Transformations, and Derived Variables

Variables with more than 15% missing values in the training cohort were excluded. Missing values in retained variables were imputed using the missForest algorithm. During resampling, imputation was re-estimated within each analysis fold.

Derived ratios were calculated after imputation of their component variables. For algorithms requiring standardized inputs, centering and scaling parameters were estimated from the relevant training data and then applied unchanged to the corresponding validation fold or internal test cohort.

Prespecified logarithmic transformations could be applied to markedly right-skewed biomarkers before standardization. The primary analysis retained the time-to-event structure and censoring information. Synthetic oversampling was omitted; weighting analyses were conducted as sensitivity analyses, with weights estimated within the training folds.

2.7. Four-Method Feature Selection

Four complementary feature-selection procedures were performed in the training cohort.

Univariable Cox regression retained 13 variables with $P < 0.05$ and hazard ratios outside the range of 0.95–1.05.

Multivariable Cox regression retained 16 variables with $P < 0.20$ after mutual adjustment. This threshold was used to preserve potentially informative predictors for subsequent modeling.

LASSO Cox regression was fitted using 10-fold cross-validation to select the penalty parameter and retained 10 variables with nonzero coefficients.

Random survival forest screening ranked predictors by variable importance and retained the top 50%, corresponding to 18 variables.

The four-way intersection yielded eight predictors: homocysteine, creatine kinase-MB, lactate dehydrogenase, gamma-glutamyl transferase, high-density lipoprotein cholesterol, venous glucose, direct bilirubin, and the CK-MB-to-total CK ratio. These variables were carried forward for model development. Complete method-specific results are provided in Supplementary Table S2.

2.8. Candidate Survival-Model Pipelines

A prespecified library of 101 survival-model pipelines was evaluated. The library included single learners and two-stage combinations involving stepwise Cox regression, LASSO, ridge regression, elastic net, CoxBoost, survival support-vector machines, supervised principal components, partial least squares Cox regression, random survival forest, and gradient boosting.

In two-stage pipelines, the first component was used for feature selection or representation learning, and the second component fitted the survival model. Supplementary Table S3 lists all 101 candidate pipelines. Model defaults, tuning parameters, and final hyperparameter settings are reported in Supplementary Table S5.

2.9. Model Comparison and Final Fitting

Candidate pipelines were compared using resampling within the training cohort. Preprocessing and feature-selection procedures were repeated within each resample.

The mean cross-validated Harrell C-index was used as the primary ranking metric. Time-dependent AUROC, AUPRC, Brier score, and calibration were used as supporting measures. Training and internal-test C-indices were reported separately.

The selected pipeline was forward stepwise Cox regression followed by random survival forest, referred to as StepCox (forward) + RSF. It was evaluated in the internal test cohort.

In the final pipeline, forward stepwise Cox regression was first applied to the eight-predictor set, after which the retained variables were entered into the RSF model. The final RSF used 1,000 trees and log-rank splitting. Predicted event probability at each horizon was calculated as one minus the estimated PSS-free probability. Candidate-pipeline performance and the basis for model selection are reported in Supplementary Table S4.

2.10. Discrimination and Prediction Error

Discrimination was evaluated using Harrell C-index and time-dependent AUROC at 1, 3, and 5 years. Time-dependent precision–recall analysis was also performed because of potential event imbalance. Prediction error was assessed using censoring-adjusted Brier scores.

The final model's internal-test performance is reported in Supplementary Table S6. Supplementary Figure S2A compares the leading candidate models, whereas Supplementary Figure S2B shows the precision–recall performance of the final model at all three prediction horizons.

2.11. Risk-Group Analysis and Classification Thresholds

For Kaplan-Meier risk-group analysis, the cutoff separating high- and low-risk patients was determined from predictions in the training cohort and then applied unchanged to the internal test cohort. PSS-free probability curves were compared using the log-rank test.

For horizon-specific binary classification summaries, the probability threshold was selected in the training cohort by maximizing the Youden index unless a clinically prespecified threshold was available. The selected threshold was then applied unchanged to the internal test cohort.

Sensitivity, specificity, accuracy, positive predictive value, negative predictive value, and F1 score were treated as secondary measures. Threshold-independent time-to-event metrics remained the primary measures of model performance. The selected thresholds are reported in Supplementary Table S11.

2.12. Calibration

Calibration was assessed separately at 1, 3, and 5 years. Calibration plots compared predicted event probabilities with censoring-adjusted observed probabilities and included the 45-degree ideal line.

Quantitative calibration measures included the calibration intercept, calibration slope, expected-to-observed ratio, Brier score, and Greenwood–Nam–D'Agostino goodness-of-fit test. Intercepts close to 0, slopes close to 1, and expected-to-observed ratios close to 1 were considered indicative of acceptable calibration. Results are reported in Supplementary Table S7.

2.13. Clinical Utility

Decision curve analysis was used to compare the net benefit of the final model with treat-all and treat-none strategies across clinically relevant threshold probabilities.

Analyses were performed separately at 1, 3, and 5 years so that the treat-all strategy reflected the event probability at each horizon. Clinical utility was indicated when the model provided greater net benefit than both reference strategies across clinically relevant threshold ranges. Threshold ranges and net-benefit results are reported in Supplementary Table S11.

2.14. Cross-Validation and Sensitivity Analyses

Five-fold and 10-fold cross-validation were performed within the training cohort as sensitivity analyses. Preprocessing, feature selection, and model fitting were repeated within each analysis fold. Performance in the held-out folds was summarized using the mean and standard deviation.

Additional sensitivity analyses included alternative fold allocations, correlation and variance-inflation-factor assessment of the final eight predictors, and comparison of weighted and unweighted survival models, when applicable. To assess the sensitivity of model performance to feature-set definition, the selected StepCox (forward) + RSF pipeline was additionally evaluated using three prespecified predictor sets: the eight-variable four-method intersection, the 27-variable union of predictors retained by at least one feature-selection method, and the complete set of 37 candidate predictors. All analyses used the same 7:3 training/internal-test split, with preprocessing and tuning conducted in the training cohort. The evaluation framework included Harrell C-index, time-dependent AUROC and AUPRC at 1, 3, and 5 years, Brier score, and calibration assessment. Sensitivity analyses are summarized in Supplementary Table S9 and Supplementary Figure S4, and the intersection-versus-union-versus-full comparison is reported in Supplementary Table S14.

2.15. Model Interpretation

Random survival forest variable importance and SHapley Additive exPlanations were used to interpret the final model.

Global SHAP importance was summarized using the mean absolute SHAP value. The beeswarm plot showed the direction and distribution of individual predictor contributions, and the decision plot showed the cumulative contributions to the model output.

Force plots for one high-risk and one low-risk patient in the internal test cohort were provided to illustrate individual prediction patterns. RSF and SHAP rankings are reported in Supplementary Table S10.

2.16. Simplified Nomogram

A simplified Cox nomogram was constructed using lactate dehydrogenase, creatine kinase-MB, and direct bilirubin. These three variables were shared by the leading RSF variable-importance and mean absolute SHAP rankings.

The full PSSRS included homocysteine and the other predictors beyond those used in the simplified nomogram. The nomogram was fitted in the training cohort and evaluated in the internal test cohort at 1, 3, and 5 years.

The simplified nomogram provided a more accessible representation of the full model using fewer predictors. Differences in time-dependent AUROC between the full PSSRS and the simplified nomogram were evaluated at 1, 3, and 5 years in the internal test cohort using paired patient-level bootstrap resampling with 1,000 replicates. Delta AUROC was defined as the AUROC of the simplified nomogram minus that of the full PSSRS, with both models evaluated on the same resampled patients in each bootstrap replicate. The corresponding delta AUROC estimates, 95% confidence intervals, and P values are reported in Supplementary Table S15.

2.17. Statistical Analysis and Uncertainty

Analyses were performed using R version 4.3.1, with Python used for SHAP visualization.

Continuous variables were summarized as median and interquartile range, and categorical variables as number and percentage. Baseline characteristics according to documented PSS status are presented in main Table 1. The training and internal test cohorts were compared descriptively using the Wilcoxon rank-sum test for continuous variables and the chi-square test or Fisher exact test for categorical variables, as appropriate; these data-split comparisons are reported in Supplementary Table S12.

All confidence intervals were two-sided 95% intervals, and $P < 0.05$ was considered statistically significant.

In the internal test cohort, patient-level bootstrap resampling with 1,000 replicates was used to estimate uncertainty where applicable. The complete metric calculation was repeated within each bootstrap sample. For paired comparisons between the full PSSRS and simplified nomogram, both models were evaluated within the

same bootstrap replicate. Cross-validation variability was summarized using the mean and standard deviation across held-out folds.

The reported performance estimates are based on internal validation. Further temporal, external, and prospective validation will be required to assess generalizability and clinical applicability. Data-access restrictions are described in the Availability of Supporting Data section.

3. Supplementary Figures

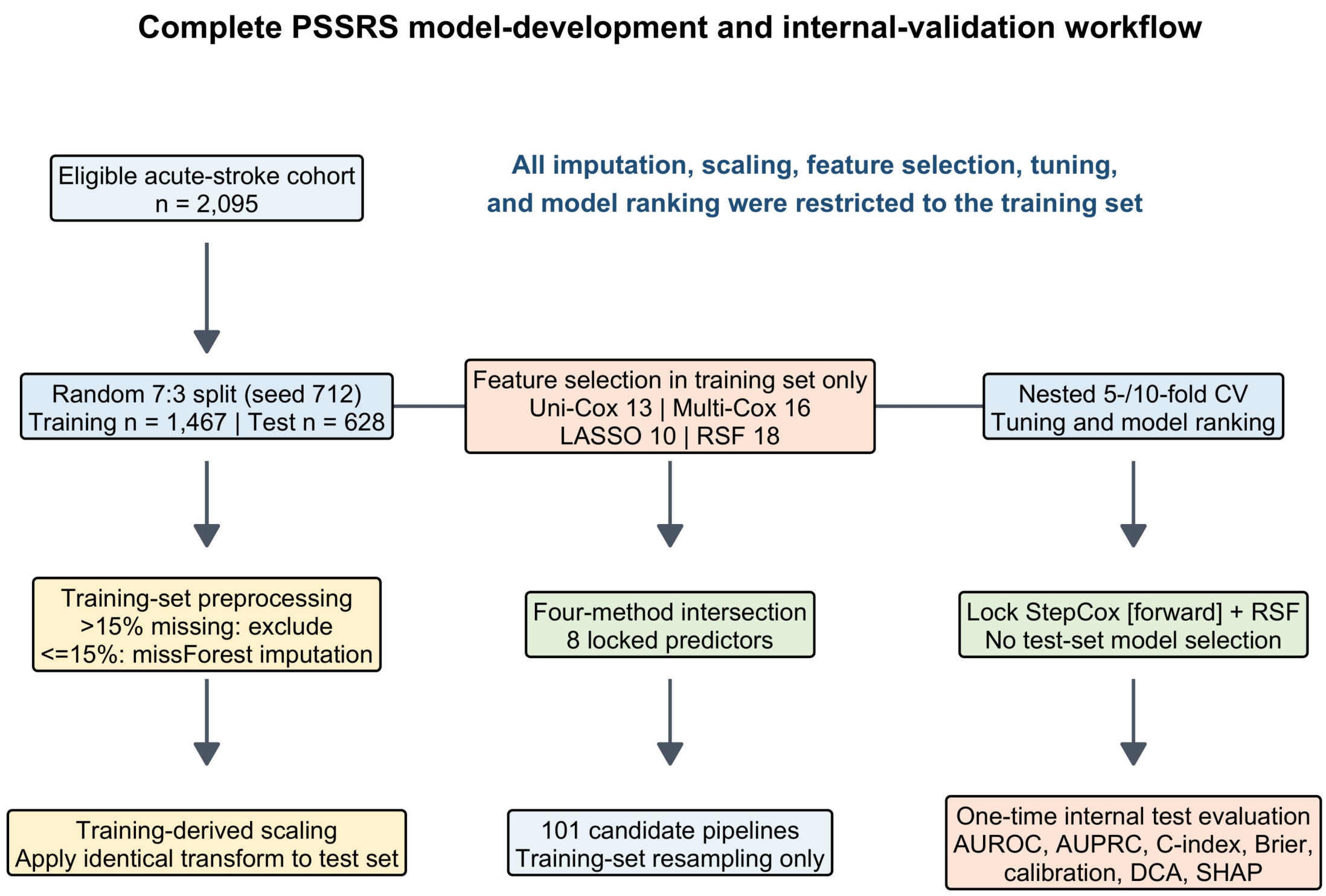

Supplementary Figure S1. Study design and model-development workflow for the PSSRS. The flow diagram shows cohort screening (2,773 records screened; 2,095 patients included), the prespecified 7:3 training/internal-test split, preprocessing and four-method feature selection in the training cohort, comparison of candidate survival-model pipelines, selection of the final model, and evaluation in the internal test cohort. The final StepCox (forward) + RSF pipeline used eight predictors: HCY, CK-MB, LDH, GGT, HDL-C, venous glucose, DBIL, and CK-MB/CK. RSF, random survival forest; HCY, homocysteine; CK-MB, creatine kinase-MB; LDH, lactate dehydrogenase; GGT, gamma-glutamyl transferase; HDL-C, high-density lipoprotein cholesterol; DBIL, direct bilirubin.

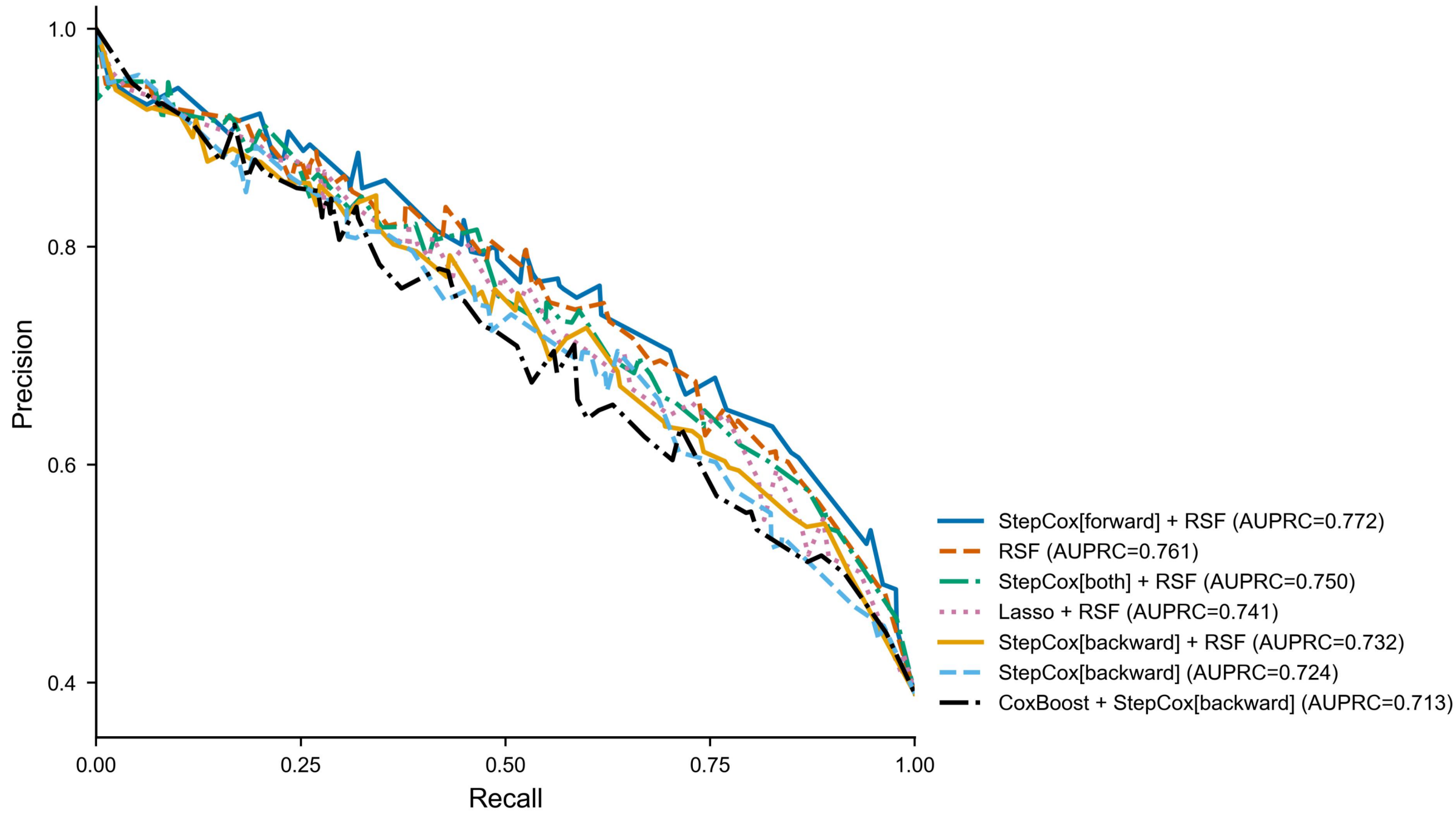

Supplementary Figure S2A. Three-year precision-recall curves for leading candidate survival-model pipelines. The curves compare the final StepCox (forward) + RSF pipeline with six leading alternatives selected for readable display. The complete set of 101 candidate pipelines and their reported performance values are provided in Supplementary Tables S3 and S4. AUPRC, area under the precision-recall curve; RSF, random survival forest.

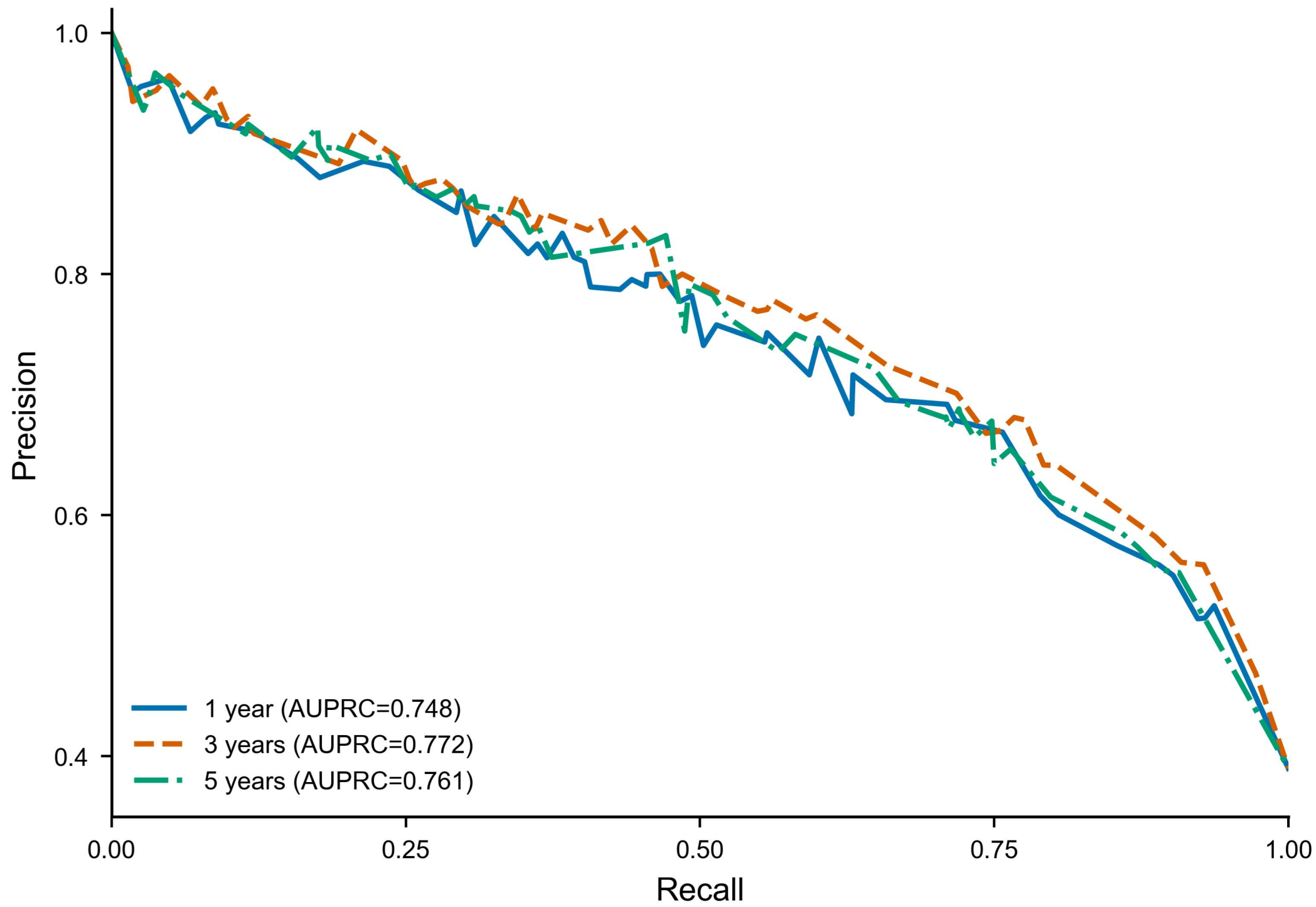

Supplementary Figure S2B. Time-specific precision-recall curves for the final PSSRS in the internal test cohort. Precision-recall curves are shown for the final model at 1, 3, and 5 years. Corresponding AUPRC values were 0.748, 0.772, and 0.761, respectively. These metrics complement the time-dependent AUROC and Brier scores reported in Supplementary Table S6. PSSRS, Post-Stroke Sequelae Risk Score; AUPRC, area under the precision-recall curve; AUROC, area under the receiver-operating-characteristic curve.

Number at risk

	Time (days) 0	400	800	1200	1600
**Training cohort (n = 1467)**					
High risk	733	324	138	14	1
Low risk	734	612	461	286	0
**Internal test cohort (n = 628)**					
High risk	391	261	165	85	0
Low risk	237	159	104	42	1
**Overall cohort (n = 2095)**					
High risk	1124	585	303	99	1
Low risk	971	771	565	328	1

Supplementary Figure S3. Numbers at risk for the Kaplan-Meier risk-group analyses. Numbers remaining at risk are shown at 0, 400, 800, 1,200, and 1,600 days for the high- and low-risk groups in the training, internal-test, and overall cohorts. The cutoff was derived in the training cohort and applied unchanged to the internal test cohort. At day 0, the high- and low-risk groups comprised 733 and 734 patients in the training cohort, 391 and 237 in the internal test cohort, and 1,124 and 971 in the overall cohort. The underlying counts are reported in Supplementary Table S8.

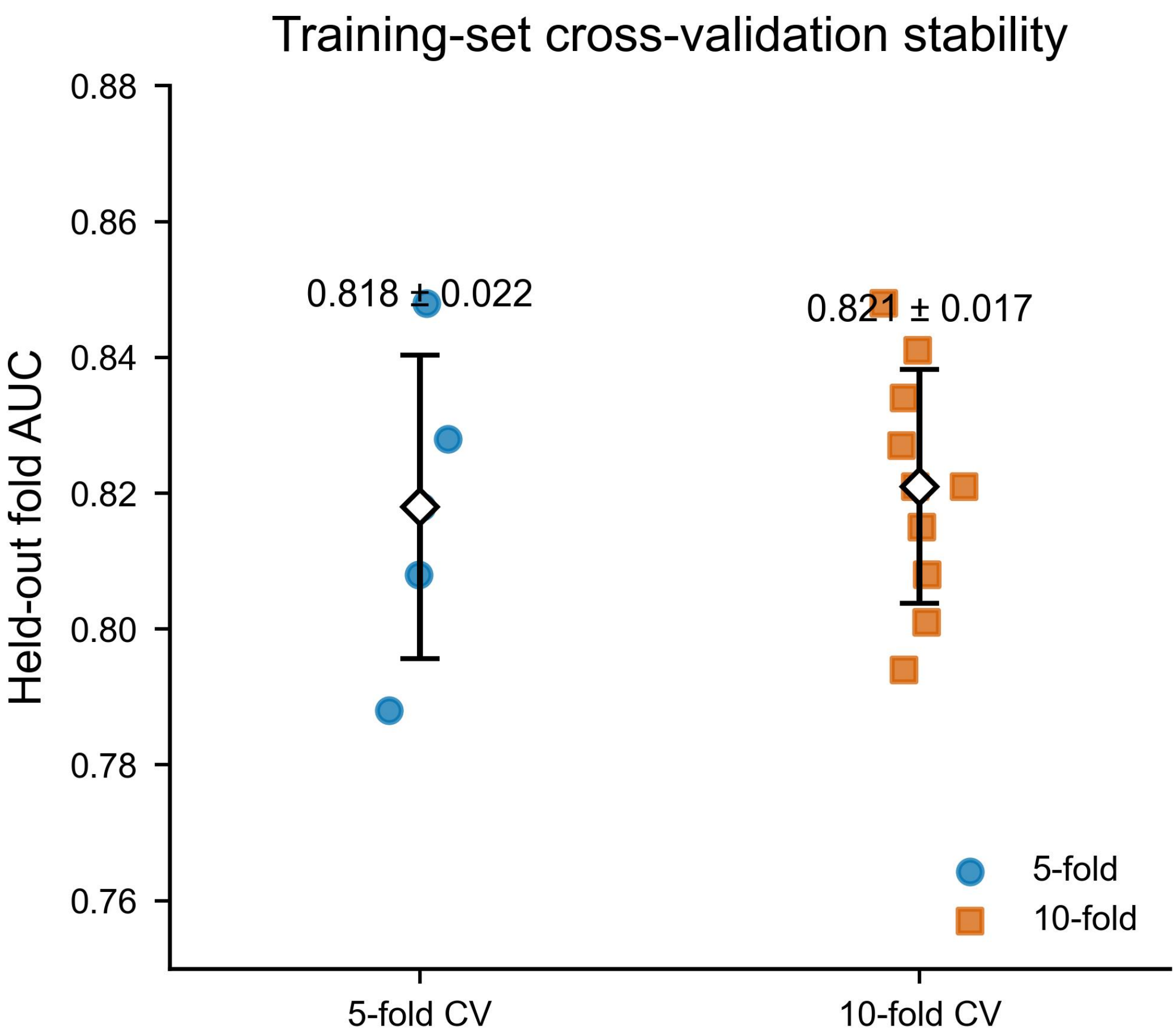

Supplementary Figure S4. Training-set cross-validation stability of the final pipeline. Fold-level held-out AUROC values are shown for fivefold and tenfold cross-validation performed within the training cohort. Open diamonds and error bars indicate mean ± standard deviation. Preprocessing and feature selection were refitted within each analysis fold. The corresponding mean AUROCs were 0.818 ± 0.022 and 0.821 ± 0.017, respectively. AUROC, area under the receiver-operating-characteristic curve.

4. Supplementary Tables

Supplementary Table S1. Candidate variables and data dictionary. The table lists the 37 candidate variables considered for model development and gives each variable's definition, unit, variable type, domain, acquisition or derivation window, missing-data rate, and final-model inclusion status.

Variable	Definition	Unit	Type	Domain	Acquisition/derivation window	Missing rate (%)	Final
TC	Total cholesterol	mmol/L	Continuous	Lipid	First eligible index-admission value, principally within 24 h	1.8	No
a-HBDH	Alpha-hydroxybutyrate dehydrogenase	U/L	Continuous	Tissue/cardiac injury	First eligible index-admission value, principally within 24 h	4.6	No
LDL-C	Low-density lipoprotein cholesterol	mmol/L	Continuous	Lipid	First eligible index-admission value, principally within 24 h	2.1	No
TBIL	Total bilirubin	μmol/L	Continuous	Hepatic	First eligible index-admission value, principally within 24 h	0.8	No
ALB/GLO	Albumin-to-globulin ratio	Ratio	Derived continuous	Protein	Derived after component-level imputation	1	No
TP(venous)	Total protein, venous	g/L	Continuous	Protein	First eligible index-admission value, principally within 24 h	0.7	No
ALB	Albumin	g/L	Continuous	Protein	First eligible index-admission value, principally within 24 h	0.6	No
IBIL	Indirect bilirubin	μmol/L	Continuous	Hepatic	First eligible index-admission value, principally within 24 h	1.2	No
LDH	Lactate dehydrogenase	U/L	Continuous	Tissue/cardiac injury	First eligible index-admission value, principally within 24 h	1.5	Yes
AST	Aspartate aminotransferase	U/L	Continuous	Hepatic	First eligible index-admission value, principally within 24 h	0.5	No
DBIL	Direct bilirubin	μmol/L	Continuous	Hepatic	First eligible index-admission value, principally within 24 h	1	Yes
Crea	Serum creatinine	μmol/L	Continuous	Renal	First eligible index-admission value, principally within 24 h	0.4	No
GLO	Globulin	g/L	Continuous	Protein	First eligible index-admission value, principally within 24 h	0.9	No
Urea	Serum urea	mmol/L	Continuous	Renal	First eligible index-admission value, principally within 24 h	0.6	No
ALT	Alanine aminotransferase	U/L	Continuous	Hepatic	First eligible index-admission value, principally within 24 h	0.5	No
m-AST	Mitochondrial aspartate aminotransferase	U/L	Continuous	Hepatic/tissue injury	First eligible index-admission value, principally within 24 h	6.8	No
eGFR	Estimated glomerular filtration rate	mL/min/1.73 m²	Derived continuous	Renal	Derived from admission renal-function measurements	0.8	No
HDL-C	High-density lipoprotein cholesterol	mmol/L	Continuous	Lipid	First eligible index-admission value, principally within 24 h	2	Yes
TG	Triglycerides	mmol/L	Continuous	Lipid	First eligible index-admission value, principally within 24 h	1.7	No
BUN/Crea	Blood urea nitrogen-to-creatinine ratio	Ratio	Derived continuous	Renal	Derived after component-level imputation	1	No
GGT	Gamma-glutamyl transferase	U/L	Continuous	Hepatic	First eligible index-admission value, principally within 24 h	0.9	Yes
LDH/a-HBDH	LDH-to-alpha-HBDH ratio	Ratio	Derived continuous	Tissue/cardiac injury	Derived after component-level imputation	4.8	No
AST/ALT	AST-to-ALT ratio	Ratio	Derived continuous	Hepatic	Derived after component-level imputation	0.7	No
HCY	Homocysteine	μmol/L	Continuous	Metabolic/vascular	First eligible index-admission value, principally within 24 h	7.4	Yes

Supplementary Table S1 (continued). Candidate variables and data dictionary.

Variable	Definition	Unit	Type	Domain	Acquisition/derivation window	Missing rate (%)	Final
AST-m/AST	Non-mitochondrial AST-to-total AST ratio, (AST − m-AST)/AST	Ratio	Derived continuous	Hepatic/tissue injury	Derived after component-level imputation	6.9	No
Ca	Serum calcium	mmol/L	Continuous	Metabolic	First eligible index-admission value, principally within 24 h	1.1	No
CK-MB	Creatine kinase-MB isoenzyme	U/L	Continuous	Cardiac injury	First eligible index-admission value, principally within 24 h	1.3	Yes
ALP	Alkaline phosphatase	U/L	Continuous	Hepatic	First eligible index-admission value, principally within 24 h	0.8	No
IMA	Ischemia-modified albumin	U/mL	Continuous	Ischemia/tissue injury	First eligible index-admission value, principally within 24 h	8.6	No
Glu(venous)	Venous plasma glucose	mmol/L	Continuous	Metabolic	First eligible index-admission value, principally within 24 h	0.6	Yes
ctCO2(B)	Calculated total carbon dioxide content in blood	mmol/L	Continuous	Acid-base	First eligible index-admission value, principally within 24 h	3.2	No
Smoking_Status	Smoking status recorded at index admission	No/Yes	Categorical	Clinical	Index admission	2.4	No
Alcohol_Status	Alcohol-use status recorded at index admission	No/Yes	Categorical	Clinical	Index admission	2.9	No
CK-MB/CK	CK-MB-to-total CK ratio	%	Derived continuous	Cardiac injury	Derived after component-level imputation	1.8	Yes
CG	Serum cholyglycine	µg/mL	Continuous	Clinical/biochemical	Index admission	5.6	No
Gender	Biological sex recorded at admission	Female/Male	Categorical	Clinical	Index admission	0	No
Age	Age at index admission	years	Continuous	Clinical	Index admission	0	No

Supplementary Table S2. Results of four feature-selection procedures. The table reports selection indicators and statistical results from univariable Cox regression, multivariable Cox regression, LASSO, and random survival forest screening. Columns include univariable and multivariable P values, hazard ratios with 95% confidence intervals, LASSO coefficients, RSF visual ranks, and membership in the four-method intersection.

Variable	Uni Cox	Uni P	Uni HR (95% CI)	Multi Cox	Multi P	Multi HR (95% CI)	LASSO selected	LASSO coefficient	RSF	RSF visual rank	Four-way intersection
TC	No	0.642	1.04 (0.89–1.21)	No	0.205	1.04 (0.93–1.16)	Yes	-0.191	No	11	No
a-HBDH	Yes	0.050	1.00 (1.00-1.00)	No	0.394	1.02 (0.90–1.15)	No	0	Yes	20	No
LDL-C	Yes	0.039	0.99 (0.94-1.10)	No	0.031	1.13 (1.03–1.23)	No	-0.012	Yes	18	No
TBIL	No	0.728	1.02 (0.89–1.17)	No	0.327	1.02 (0.86–1.21)	No	0	Yes	28	No
ALB/GLO	No	0.769	0.98 (0.86–1.11)	No	0.386	0.97 (0.83–1.14)	No	0	No	9	No
TP(venous)	No	0.870	0.97 (0.84–1.12)	Yes	0.155	0.98 (0.98-0.98)	No	0	No	33	No
ALB	No	0.200	0.95 (0.85–1.06)	No	0.619	0.95 (0.82–1.10)	No	0	No	19	No
IBIL	No	0.771	1.03 (0.92–1.15)	No	0.352	1.03 (0.88–1.19)	No	0	No	21	No
LDH	Yes	0.002	2.11 (1.83-2.39)	Yes	0.012	1.10 (1.05-1.16)	Yes	0.552	Yes	1	Yes
AST	No	0.023	1.16 (1.05–1.28)	No	0.448	1.05 (0.89–1.23)	No	0	Yes	26	No
DBIL	Yes	0.008	1.52 (1.32-1.73)	Yes	0.153	0.98 (0.97-0.98)	Yes	0.298	Yes	3	Yes
Crea	No	0.544	1.05 (0.92–1.19)	No	0.024	1.23 (1.12–1.34)	No	0	Yes	15	No
GLO	No	0.483	1.05 (0.89–1.25)	No	0.064	1.05 (0.91–1.22)	No	0	No	31	No
Urea	No	0.470	1.03 (0.92–1.16)	No	0.037	1.19 (1.06–1.33)	No	0	Yes	29	No
ALT	Yes	0.047	1.06 (1.00-1.10)	Yes	0.051	1.12 (1.10-1.15)	No	0	No	24	No
m-AST	No	0.501	1.02 (0.87–1.21)	Yes	0.062	1.00 (1.00-1.00)	No	0	No	10	No
eGFR	No	0.038	0.87 (0.80–0.96)	Yes	0.038	1.08 (1.03-1.13)	No	0.015	Yes	12	No
HDL-C	Yes	0.011	0.83 (0.71-0.95)	Yes	0.062	1.10 (1.09-1.11)	Yes	-0.290	Yes	8	Yes
TG	No	0.726	1.03 (0.91–1.17)	No	0.289	1.03 (0.89–1.20)	Yes	0.166	No	13	No
BUN/Crea	No	0.094	1.02 (0.88–1.19)	No	0.477	1.02 (0.92–1.14)	No	0	No	17	No
GGT	Yes	0.016	1.35 (1.17-1.54)	Yes	0.087	1.01 (1.00-1.03)	Yes	0.211	Yes	6	Yes
LDH/a-HBDH	No	0.086	1.05 (0.95–1.18)	Yes	0.098	0.56 (0.54-0.58)	No	0	No	37	No
AST/ALT	No	0.467	1.05 (0.89–1.25)	No	0.868	1.05 (0.91–1.21)	No	0	No	25	No
HCY	Yes	0.042	1.28 (1.12-1.44)	Yes	0.188	1.00 (1.00-1.00)	Yes	0.233	Yes	5	Yes

Supplementary Table S2 (continued). Results of four feature-selection procedures.

Variable	Uni Cox	Uni P	Uni HR (95% CI)	Multi Cox	Multi P	Multi HR (95% CI)	LASSO selected	LASSO coefficient	RSF	RSF visual rank	Four-way intersection
AST-m/AST	No	0.188	1.02 (0.88–1.20)	No	0.607	1.02 (0.90–1.16)	No	0	No	14	No
Ca	Yes	0.038	1.60 (1.00-2.40)	Yes	0.017	1.80 (1.80-1.80)	No	-0.020	No	22	No
CK-MB	Yes	0.027	1.55 (1.37-1.73)	Yes	0.114	1.03 (1.02-1.03)	Yes	0.363	Yes	2	Yes
ALP	No	0.298	1.02 (0.89–1.17)	No	0.681	1.03 (0.87–1.21)	No	0	No	23	No
IMA	Yes	0.048	1.20 (0.99-1.40)	No	0.029	1.21 (1.09–1.34)	No	0	Yes	34	No
Glu(venous)	Yes	0.044	1.33 (1.15-1.51)	Yes	0.041	1.03 (1.01-1.06)	Yes	0.258	Yes	7	Yes
ctCO2(B)	No	0.079	1.04 (0.88–1.22)	No	0.480	1.04 (0.90–1.19)	No	0	Yes	36	No
Smoking_Status	No	0.021	1.15 (1.06–1.25)	No	0.040	1.09 (1.02–1.17)	No	0	No	35	No
Alcohol_Status	No	0.106	1.03 (0.91–1.18)	No	0.489	1.04 (0.89–1.21)	No	0	No	16	No
CK-MB/CK	Yes	0.032	1.10 (0.99-1.20)	Yes	0.112	1.04 (1.03-1.05)	Yes	0.254	Yes	4	Yes
CG	No	0.663	1.02 (0.87–1.20)	Yes	0.084	0.98 (0.98-0.98)	No	0	No	27	No
Gender	No	0.754	1.06 (0.94–1.19)	Yes	0.058	0.89 (0.88-0.89)	No	-0.012	No	32	No
Age	No	0.040	1.19 (1.06–1.32)	No	0.016	1.13 (1.03–1.24)	No	0	Yes	30	No

Supplementary Table S3. Candidate modeling pipelines. The table lists the 101 pipeline IDs, model pipelines, feature-selection steps, final learners, script-level defaults, and evaluation contexts used during model development.

Pipeline ID	Model pipeline	Feature-selection step	Final learner	Script-level defaults	Evaluation context
1	Lasso + StepCox [both]	Lasso	StepCox [both]	alpha=1; lambda selected by 10-fold CV (lambda.min) | AIC-based stepwise Cox; direction=both	Training-cohort tuning; internal-test evaluation
2	survival-SVM	None / embedded	survival-SVM	gamma.mu=1; opt.meth=ipop	Training-cohort tuning; internal-test evaluation
3	CoxBoost + survival-SVM	CoxBoost	survival-SVM	penalty optimized; maxstepno=500; 10-fold CV; type=verweij | gamma.mu=1; opt.meth=ipop	Training-cohort tuning; internal-test evaluation
4	Ridge	None / embedded	Ridge	alpha=0; lambda selected by 10-fold CV	Training-cohort tuning; internal-test evaluation
5	Lasso + survival-SVM	Lasso	survival-SVM	alpha=1; lambda selected by 10-fold CV (lambda.min) | gamma.mu=1; opt.meth=ipop	Training-cohort tuning; internal-test evaluation
6	SuperPC	None / embedded	SuperPC	20 thresholds; 10-fold CV; 3 components; min.features=5	Training-cohort tuning; internal-test evaluation
7	CoxBoost + Ridge	CoxBoost	Ridge	penalty optimized; maxstepno=500; 10-fold CV; type=verweij | alpha=0; lambda selected by 10-fold CV	Training-cohort tuning; internal-test evaluation
8	Enet[a=0.1]	None / embedded	Enet [alpha=0.1]	alpha=0.1; lambda selected by 10-fold CV	Training-cohort tuning; internal-test evaluation
9	CoxBoost + Enet[a=0.1]	CoxBoost	Enet [alpha=0.1]	penalty optimized; maxstepno=500; 10-fold CV; type=verweij | alpha=0.1; lambda selected by 10-fold CV	Training-cohort tuning; internal-test evaluation
10	Enet[a=0.2]	None / embedded	Enet [alpha=0.2]	alpha=0.2; lambda selected by 10-fold CV	Training-cohort tuning; internal-test evaluation
11	Enet[a=0.3]	None / embedded	Enet [alpha=0.3]	alpha=0.3; lambda selected by 10-fold CV	Training-cohort tuning; internal-test evaluation
12	CoxBoost + Enet[a=0.3]	CoxBoost	Enet [alpha=0.3]	penalty optimized; maxstepno=500; 10-fold CV; type=verweij | alpha=0.3; lambda selected by 10-fold CV	Training-cohort tuning; internal-test evaluation
13	CoxBoost + Enet[a=0.2]	CoxBoost	Enet [alpha=0.2]	penalty optimized; maxstepno=500; 10-fold CV; type=verweij | alpha=0.2; lambda selected by 10-fold CV	Training-cohort tuning; internal-test evaluation
14	Enet[a=0.4]	None / embedded	Enet [alpha=0.4]	alpha=0.4; lambda selected by 10-fold CV	Training-cohort tuning; internal-test evaluation
15	CoxBoost + Enet[a=0.4]	CoxBoost	Enet [alpha=0.4]	penalty optimized; maxstepno=500; 10-fold CV; type=verweij | alpha=0.4; lambda selected by 10-fold CV	Training-cohort tuning; internal-test evaluation
16	Lasso + CoxBoost	Lasso	CoxBoost	alpha=1; lambda selected by 10-fold CV (lambda.min) | penalty optimized; maxstepno=500; 10-fold CV; type=verweij	Training-cohort tuning; internal-test evaluation
17	Enet[a=0.5]	None / embedded	Enet [alpha=0.5]	alpha=0.5; lambda selected by 10-fold CV	Training-cohort tuning; internal-test evaluation
18	CoxBoost	None / embedded	CoxBoost	penalty optimized; maxstepno=500; 10-fold CV; type=verweij	Training-cohort tuning; internal-test evaluation

Supplementary Table S3 (continued). Candidate modeling pipelines.

Pipeline ID	Model pipeline	Feature-selection step	Final learner	Script-level defaults	Evaluation context
19	CoxBoost + Enet[a=0.5]	CoxBoost	Enet [alpha=0.5]	penalty optimized; maxstepno=500; 10-fold CV; type=verweij | alpha=0.5; lambda selected by 10-fold CV	Training-cohort tuning; internal-test evaluation
20	Enet[a=0.6]	None / embedded	Enet [alpha=0.6]	alpha=0.6; lambda selected by 10-fold CV	Training-cohort tuning; internal-test evaluation
21	CoxBoost + Enet[a=0.6]	CoxBoost	Enet [alpha=0.6]	penalty optimized; maxstepno=500; 10-fold CV; type=verweij | alpha=0.6; lambda selected by 10-fold CV	Training-cohort tuning; internal-test evaluation
22	CoxBoost + Enet[a=0.7]	CoxBoost	Enet [alpha=0.7]	penalty optimized; maxstepno=500; 10-fold CV; type=verweij | alpha=0.7; lambda selected by 10-fold CV	Training-cohort tuning; internal-test evaluation
23	CoxBoost + Enet[a=0.8]	CoxBoost	Enet [alpha=0.8]	penalty optimized; maxstepno=500; 10-fold CV; type=verweij | alpha=0.8; lambda selected by 10-fold CV	Training-cohort tuning; internal-test evaluation
24	Enet[a=0.8]	None / embedded	Enet [alpha=0.8]	alpha=0.8; lambda selected by 10-fold CV	Training-cohort tuning; internal-test evaluation
25	Enet[a=0.9]	None / embedded	Enet [alpha=0.9]	alpha=0.9; lambda selected by 10-fold CV	Training-cohort tuning; internal-test evaluation
26	Lasso	None / embedded	Lasso	alpha=1; lambda selected by 10-fold CV (lambda.min)	Training-cohort tuning; internal-test evaluation
27	Enet[a=0.7]	None / embedded	Enet [alpha=0.7]	alpha=0.7; lambda selected by 10-fold CV	Training-cohort tuning; internal-test evaluation
28	CoxBoost + Enet[a=0.9]	CoxBoost	Enet [alpha=0.9]	penalty optimized; maxstepno=500; 10-fold CV; type=verweij | alpha=0.9; lambda selected by 10-fold CV	Training-cohort tuning; internal-test evaluation
29	CoxBoost + Lasso	CoxBoost	Lasso	penalty optimized; maxstepno=500; 10-fold CV; type=verweij | alpha=1; lambda selected by 10-fold CV (lambda.min)	Training-cohort tuning; internal-test evaluation
30	Lasso + plsRcox	Lasso	plsRcox	alpha=1; lambda selected by 10-fold CV (lambda.min) | 10-component cross-validation; sparse=TRUE	Training-cohort tuning; internal-test evaluation
31	CoxBoost + plsRcox	CoxBoost	plsRcox	penalty optimized; maxstepno=500; 10-fold CV; type=verweij | 10-component cross-validation; sparse=TRUE	Training-cohort tuning; internal-test evaluation
32	CoxBoost + StepCox [forward]	CoxBoost	StepCox [forward]	penalty optimized; maxstepno=500; 10-fold CV; type=verweij | AIC-based stepwise Cox; direction=forward	Training-cohort tuning; internal-test evaluation
33	Lasso + StepCox [forward]	Lasso	StepCox [forward]	alpha=1; lambda selected by 10-fold CV (lambda.min) | AIC-based stepwise Cox; direction=forward	Training-cohort tuning; internal-test evaluation
34	RSF + survival-SVM	RSF	survival-SVM	ntree=1000; nodesize=5; mtry=3; nsplit=10; sampling fraction=0.632	Training-cohort tuning; internal-test evaluation
35	CoxBoost + SuperPC	CoxBoost	SuperPC	penalty optimized; maxstepno=500; 10-fold CV; type=verweij | 20 thresholds; 10-fold CV; 3 components; min.features=5	Training-cohort tuning; internal-test evaluation
36	StepCox [forward]	None / embedded	StepCox [forward]	AIC-based stepwise Cox; direction=forward	Training-cohort tuning; internal-test evaluation

Supplementary Table S3 (continued). Candidate modeling pipelines.

Pipeline ID	Model pipeline	Feature-selection step	Final learner	Script-level defaults	Evaluation context
37	plsRcox	None / embedded	plsRcox	10-component cross-validation; sparse=TRUE	Training-cohort tuning; internal-test evaluation
38	RSF + Ridge	RSF	Ridge	ntree=1000; nodesize=5; mtry=3; nsplit=10; sampling fraction=0.632	Training-cohort tuning; internal-test evaluation
39	RSF + Enet[a=0.1]	RSF	Enet [alpha=0.1]	ntree=1000; nodesize=5; mtry=3; nsplit=10; sampling fraction=0.632	Training-cohort tuning; internal-test evaluation
40	Lasso + SuperPC	Lasso	SuperPC	alpha=1; lambda selected by 10-fold CV (lambda.min) | 20 thresholds; 10-fold CV; 3 components; min.features=5	Training-cohort tuning; internal-test evaluation
41	RSF + plsRcox	RSF	plsRcox	ntree=1000; nodesize=5; mtry=3; nsplit=10; sampling fraction=0.632	Training-cohort tuning; internal-test evaluation
42	StepCox [forward] + RSF	StepCox [forward]	RSF	ntree=1000; nodesize=5; mtry=3; nsplit=10; sampling fraction=0.632	Training-cohort tuning; internal-test evaluation
43	RSF + Enet[a=0.2]	RSF	Enet [alpha=0.2]	ntree=1000; nodesize=5; mtry=3; nsplit=10; sampling fraction=0.632	Training-cohort tuning; internal-test evaluation
44	RSF + Enet[a=0.3]	RSF	Enet [alpha=0.3]	ntree=1000; nodesize=5; mtry=3; nsplit=10; sampling fraction=0.632	Training-cohort tuning; internal-test evaluation
45	RSF + Enet[a=0.6]	RSF	Enet [alpha=0.6]	ntree=1000; nodesize=5; mtry=3; nsplit=10; sampling fraction=0.632	Training-cohort tuning; internal-test evaluation
46	RSF + Lasso	RSF	Lasso	ntree=1000; nodesize=5; mtry=3; nsplit=10; sampling fraction=0.632	Training-cohort tuning; internal-test evaluation
47	RSF + Enet[a=0.7]	RSF	Enet [alpha=0.7]	ntree=1000; nodesize=5; mtry=3; nsplit=10; sampling fraction=0.632	Training-cohort tuning; internal-test evaluation
48	RSF + Enet[a=0.5]	RSF	Enet [alpha=0.5]	ntree=1000; nodesize=5; mtry=3; nsplit=10; sampling fraction=0.632	Training-cohort tuning; internal-test evaluation
49	RSF + CoxBoost	RSF	CoxBoost	ntree=1000; nodesize=5; mtry=3; nsplit=10; sampling fraction=0.632	Training-cohort tuning; internal-test evaluation
50	RSF + Enet[a=0.9]	RSF	Enet [alpha=0.9]	ntree=1000; nodesize=5; mtry=3; nsplit=10; sampling fraction=0.632	Training-cohort tuning; internal-test evaluation
51	RSF + Enet[a=0.4]	RSF	Enet [alpha=0.4]	ntree=1000; nodesize=5; mtry=3; nsplit=10; sampling fraction=0.632	Training-cohort tuning; internal-test evaluation
52	RSF + Enet[a=0.8]	RSF	Enet [alpha=0.8]	ntree=1000; nodesize=5; mtry=3; nsplit=10; sampling fraction=0.632	Training-cohort tuning; internal-test evaluation
53	RSF + StepCox [both]	RSF	StepCox [both]	ntree=1000; nodesize=5; mtry=3; nsplit=10; sampling fraction=0.632	Training-cohort tuning; internal-test evaluation
54	RSF + StepCox [backward]	RSF	StepCox [backward]	ntree=1000; nodesize=5; mtry=3; nsplit=10; sampling fraction=0.632	Training-cohort tuning; internal-test evaluation

Supplementary Table S3 (continued). Candidate modeling pipelines.

Pipeline ID	Model pipeline	Feature-selection step	Final learner	Script-level defaults	Evaluation context
55	StepCox [both] + Ridge	StepCox [both]	Ridge	AIC-based stepwise Cox; direction=both | alpha=0; lambda selected by 10-fold CV	Training-cohort tuning; internal-test evaluation
56	StepCox [backward] + Ridge	StepCox [backward]	Ridge	AIC-based stepwise Cox; direction=backward | alpha=0; lambda selected by 10-fold CV	Training-cohort tuning; internal-test evaluation
57	StepCox [both] + plsRcox	StepCox [both]	plsRcox	AIC-based stepwise Cox; direction=both | 10-component cross-validation; sparse=TRUE	Training-cohort tuning; internal-test evaluation
58	StepCox [backward] + plsRcox	StepCox [backward]	plsRcox	AIC-based stepwise Cox; direction=backward | 10-component cross-validation; sparse=TRUE	Training-cohort tuning; internal-test evaluation
59	StepCox [both] + Enet[a=0.9]	StepCox [both]	Enet [alpha=0.9]	AIC-based stepwise Cox; direction=both | alpha=0.9; lambda selected by 10-fold CV	Training-cohort tuning; internal-test evaluation
60	StepCox [backward] + Enet[a=0.9]	StepCox [backward]	Enet [alpha=0.9]	AIC-based stepwise Cox; direction=backward | alpha=0.9; lambda selected by 10-fold CV	Training-cohort tuning; internal-test evaluation
61	StepCox [both] + Enet[a=0.1]	StepCox [both]	Enet [alpha=0.1]	AIC-based stepwise Cox; direction=both | alpha=0.1; lambda selected by 10-fold CV	Training-cohort tuning; internal-test evaluation
62	StepCox [backward] + Enet[a=0.1]	StepCox [backward]	Enet [alpha=0.1]	AIC-based stepwise Cox; direction=backward | alpha=0.1; lambda selected by 10-fold CV	Training-cohort tuning; internal-test evaluation
63	StepCox [both] + Enet[a=0.8]	StepCox [both]	Enet [alpha=0.8]	AIC-based stepwise Cox; direction=both | alpha=0.8; lambda selected by 10-fold CV	Training-cohort tuning; internal-test evaluation
64	StepCox [backward] + Enet[a=0.8]	StepCox [backward]	Enet [alpha=0.8]	AIC-based stepwise Cox; direction=backward | alpha=0.8; lambda selected by 10-fold CV	Training-cohort tuning; internal-test evaluation
65	StepCox [both] + Enet[a=0.2]	StepCox [both]	Enet [alpha=0.2]	AIC-based stepwise Cox; direction=both | alpha=0.2; lambda selected by 10-fold CV	Training-cohort tuning; internal-test evaluation
66	StepCox [backward] + Enet[a=0.2]	StepCox [backward]	Enet [alpha=0.2]	AIC-based stepwise Cox; direction=backward | alpha=0.2; lambda selected by 10-fold CV	Training-cohort tuning; internal-test evaluation
67	StepCox [both] + Lasso	StepCox [both]	Lasso	AIC-based stepwise Cox; direction=both | alpha=1; lambda selected by 10-fold CV (lambda.min)	Training-cohort tuning; internal-test evaluation
68	StepCox [backward] + Lasso	StepCox [backward]	Lasso	AIC-based stepwise Cox; direction=backward | alpha=1; lambda selected by 10-fold CV (lambda.min)	Training-cohort tuning; internal-test evaluation
69	StepCox [both] + Enet[a=0.6]	StepCox [both]	Enet [alpha=0.6]	AIC-based stepwise Cox; direction=both | alpha=0.6; lambda selected by 10-fold CV	Training-cohort tuning; internal-test evaluation
70	StepCox [backward] + Enet[a=0.6]	StepCox [backward]	Enet [alpha=0.6]	AIC-based stepwise Cox; direction=backward | alpha=0.6; lambda selected by 10-fold CV	Training-cohort tuning; internal-test evaluation
71	CoxBoost + GBM	CoxBoost	GBM	penalty optimized; maxstepno=500; 10-fold CV; type=verweij | n.trees<=10000; depth=3; minobs=10; shrinkage=0.001; 10-fold CV	Training-cohort tuning; internal-test evaluation
72	StepCox [both] + Enet[a=0.7]	StepCox [both]	Enet [alpha=0.7]	AIC-based stepwise Cox; direction=both | alpha=0.7; lambda selected by 10-fold CV	Training-cohort tuning; internal-test evaluation

Supplementary Table S3 (continued). Candidate modeling pipelines.

Pipeline ID	Model pipeline	Feature-selection step	Final learner	Script-level defaults	Evaluation context
73	StepCox [backward] + Enet[a=0.7]	StepCox [backward]	Enet [alpha=0.7]	AIC-based stepwise Cox; direction=backward | alpha=0.7; lambda selected by 10-fold CV	Training-cohort tuning; internal-test evaluation
74	Lasso + StepCox [backward]	Lasso	StepCox [backward]	alpha=1; lambda selected by 10-fold CV (lambda.min) | AIC-based stepwise Cox; direction=backward	Training-cohort tuning; internal-test evaluation
75	StepCox [both]	None / embedded	StepCox [both]	AIC-based stepwise Cox; direction=both	Training-cohort tuning; internal-test evaluation
76	StepCox [backward]	None / embedded	StepCox [backward]	AIC-based stepwise Cox; direction=backward	Training-cohort tuning; internal-test evaluation
77	CoxBoost + StepCox [both]	CoxBoost	StepCox [both]	penalty optimized; maxstepno=500; 10-fold CV; type=verweij | AIC-based stepwise Cox; direction=both	Training-cohort tuning; internal-test evaluation
78	CoxBoost + StepCox [backward]	CoxBoost	StepCox [backward]	penalty optimized; maxstepno=500; 10-fold CV; type=verweij | AIC-based stepwise Cox; direction=backward	Training-cohort tuning; internal-test evaluation
79	StepCox [both] + Enet[a=0.4]	StepCox [both]	Enet [alpha=0.4]	AIC-based stepwise Cox; direction=both | alpha=0.4; lambda selected by 10-fold CV	Training-cohort tuning; internal-test evaluation
80	StepCox [backward] + Enet[a=0.4]	StepCox [backward]	Enet [alpha=0.4]	AIC-based stepwise Cox; direction=backward | alpha=0.4; lambda selected by 10-fold CV	Training-cohort tuning; internal-test evaluation
81	StepCox [both] + Enet[a=0.3]	StepCox [both]	Enet [alpha=0.3]	AIC-based stepwise Cox; direction=both | alpha=0.3; lambda selected by 10-fold CV	Training-cohort tuning; internal-test evaluation
82	StepCox [backward] + Enet[a=0.3]	StepCox [backward]	Enet [alpha=0.3]	AIC-based stepwise Cox; direction=backward | alpha=0.3; lambda selected by 10-fold CV	Training-cohort tuning; internal-test evaluation
83	StepCox [both] + CoxBoost	StepCox [both]	CoxBoost	AIC-based stepwise Cox; direction=both | penalty optimized; maxstepno=500; 10-fold CV; type=verweij	Training-cohort tuning; internal-test evaluation
84	StepCox [backward] + CoxBoost	StepCox [backward]	CoxBoost	AIC-based stepwise Cox; direction=backward | penalty optimized; maxstepno=500; 10-fold CV; type=verweij	Training-cohort tuning; internal-test evaluation
85	StepCox [both] + Enet[a=0.5]	StepCox [both]	Enet [alpha=0.5]	AIC-based stepwise Cox; direction=both | alpha=0.5; lambda selected by 10-fold CV	Training-cohort tuning; internal-test evaluation
86	StepCox [backward] + Enet[a=0.5]	StepCox [backward]	Enet [alpha=0.5]	AIC-based stepwise Cox; direction=backward | alpha=0.5; lambda selected by 10-fold CV	Training-cohort tuning; internal-test evaluation
87	CoxBoost + RSF	CoxBoost	RSF	ntree=1000; nodesize=5; mtry=3; nsplit=10; sampling fraction=0.632	Training-cohort tuning; internal-test evaluation
88	RSF + SuperPC	RSF	SuperPC	ntree=1000; nodesize=5; mtry=3; nsplit=10; sampling fraction=0.632	Training-cohort tuning; internal-test evaluation
89	RSF	None / embedded	RSF	ntree=1000; nodesize=5; mtry=3; nsplit=10; sampling fraction=0.632	Training-cohort tuning; internal-test evaluation
90	Lasso + GBM	Lasso	GBM	alpha=1; lambda selected by 10-fold CV (lambda.min) | n.trees<=10000; depth=3; minobs=10; shrinkage=0.001; 10-fold CV	Training-cohort tuning; internal-test evaluation

Supplementary Table S3 (continued). Candidate modeling pipelines.

Pipeline ID	Model pipeline	Feature-selection step	Final learner	Script-level defaults	Evaluation context
91	RSF + GBM	RSF	GBM	ntree=1000; nodesize=5; mtry=3; nsplit=10; sampling fraction=0.632	Training-cohort tuning; internal-test evaluation
92	GBM	None / embedded	GBM	n.trees<=10000; depth=3; minobs=10; shrinkage=0.001; 10-fold CV	Training-cohort tuning; internal-test evaluation
93	StepCox [both] + survival-SVM	StepCox [both]	survival-SVM	AIC-based stepwise Cox; direction=both | gamma.mu=1; opt.meth=ipop	Training-cohort tuning; internal-test evaluation
94	StepCox [backward] + survival-SVM	StepCox [backward]	survival-SVM	AIC-based stepwise Cox; direction=backward | gamma.mu=1; opt.meth=ipop	Training-cohort tuning; internal-test evaluation
95	Lasso + RSF	Lasso	RSF	ntree=1000; nodesize=5; mtry=3; nsplit=10; sampling fraction=0.632	Training-cohort tuning; internal-test evaluation
96	StepCox [both] + GBM	StepCox [both]	GBM	AIC-based stepwise Cox; direction=both | n.trees<=10000; depth=3; minobs=10; shrinkage=0.001; 10-fold CV	Training-cohort tuning; internal-test evaluation
97	StepCox [backward] + GBM	StepCox [backward]	GBM	AIC-based stepwise Cox; direction=backward | n.trees<=10000; depth=3; minobs=10; shrinkage=0.001; 10-fold CV	Training-cohort tuning; internal-test evaluation
98	StepCox [both] + RSF	StepCox [both]	RSF	ntree=1000; nodesize=5; mtry=3; nsplit=10; sampling fraction=0.632	Training-cohort tuning; internal-test evaluation
99	StepCox [backward] + RSF	StepCox [backward]	RSF	ntree=1000; nodesize=5; mtry=3; nsplit=10; sampling fraction=0.632	Training-cohort tuning; internal-test evaluation
100	StepCox [both] + SuperPC	StepCox [both]	SuperPC	AIC-based stepwise Cox; direction=both | 20 thresholds; 10-fold CV; 3 components; min.features=5	Training-cohort tuning; internal-test evaluation
101	StepCox [backward] + SuperPC	StepCox [backward]	SuperPC	AIC-based stepwise Cox; direction=backward | 20 thresholds; 10-fold CV; 3 components; min.features=5	Training-cohort tuning; internal-test evaluation

Supplementary Table S4. Performance ranking of candidate modeling pipelines. The table orders the 101 pipelines by heatmap display rank and reports training C-index, internal-test C-index, train-test C-index gap, 1-, 3-, and 5-year AUROC, and 1-, 3-, and 5-year AUPRC.

Pipeline ID	Heatmap display rank	Model	Train C-index	Test C-index	Train−test gap	1-y AUROC	3-y AUROC	5-y AUROC	1-y AUPRC	3-y AUPRC	5-y AUPRC
42	1	StepCox [forward] + RSF	0.870	0.850	0.020	0.800	0.830	0.820	0.748	0.772	0.761
89	2	RSF	0.870	0.840	0.030	0.793	0.821	0.812	0.741	0.763	0.753
75	3	StepCox [both]	0.860	0.830	0.030	0.779	0.807	0.798	0.727	0.749	0.739
98	4	StepCox [both] + RSF	0.860	0.830	0.030	0.786	0.814	0.806	0.734	0.756	0.747
99	5	StepCox [backward] + RSF	0.850	0.830	0.020	0.772	0.799	0.791	0.720	0.741	0.732
76	6	StepCox [backward]	0.840	0.820	0.020	0.765	0.791	0.784	0.713	0.733	0.725
78	7	CoxBoost + StepCox [backward]	0.840	0.820	0.020	0.756	0.784	0.776	0.704	0.726	0.717
36	8	StepCox [forward]	0.830	0.810	0.020	0.792	0.817	0.808	0.740	0.759	0.749
95	9	Lasso + RSF	0.830	0.810	0.020	0.780	0.805	0.796	0.728	0.747	0.737
32	10	CoxBoost + StepCox [forward]	0.830	0.800	0.030	0.778	0.803	0.794	0.726	0.745	0.735
59	11	StepCox [both] + Enet[a=0.9]	0.830	0.800	0.030	0.778	0.803	0.794	0.726	0.745	0.735
77	12	CoxBoost + StepCox [both]	0.830	0.800	0.030	0.774	0.799	0.790	0.722	0.741	0.731
14	13	Enet[a=0.4]	0.820	0.800	0.020	0.771	0.796	0.787	0.719	0.738	0.728
15	14	CoxBoost + Enet[a=0.4]	0.820	0.800	0.020	0.785	0.810	0.801	0.733	0.752	0.742
60	15	StepCox [backward] + Enet[a=0.9]	0.820	0.800	0.020	0.777	0.802	0.793	0.725	0.744	0.734
63	16	StepCox [both] + Enet[a=0.8]	0.810	0.790	0.020	0.756	0.781	0.772	0.704	0.723	0.713
64	17	StepCox [backward] + Enet[a=0.8]	0.810	0.790	0.020	0.757	0.782	0.773	0.705	0.724	0.714
87	18	CoxBoost + RSF	0.811	0.789	0.022	0.765	0.790	0.781	0.713	0.732	0.722
8	19	Enet[a=0.1]	0.810	0.780	0.030	0.759	0.784	0.775	0.707	0.726	0.716
72	20	StepCox [both] + Enet[a=0.7]	0.810	0.780	0.030	0.753	0.778	0.769	0.701	0.720	0.710
73	21	StepCox [backward] + Enet[a=0.7]	0.810	0.780	0.030	0.746	0.771	0.762	0.694	0.713	0.703
9	22	CoxBoost + Enet[a=0.1]	0.800	0.780	0.020	0.755	0.780	0.771	0.703	0.722	0.712
11	23	Enet[a=0.3]	0.800	0.780	0.020	0.756	0.781	0.772	0.704	0.723	0.713
12	24	CoxBoost + Enet[a=0.3]	0.800	0.770	0.030	0.748	0.773	0.764	0.696	0.715	0.705

Supplementary Table S4 (continued). Performance ranking of candidate modeling pipelines.

Pipeline ID	Heatmap display rank	Model	Train C-index	Test C-index	Train−test gap	1-y AUROC	3-y AUROC	5-y AUROC	1-y AUPRC	3-y AUPRC	5-y AUPRC
17	25	Enet[a=0.5]	0.800	0.770	0.030	0.751	0.776	0.767	0.699	0.718	0.708
69	26	StepCox [both] + Enet[a=0.6]	0.800	0.770	0.030	0.754	0.779	0.770	0.702	0.721	0.711
70	27	StepCox [backward] + Enet[a=0.6]	0.800	0.770	0.030	0.745	0.770	0.761	0.693	0.712	0.702
10	28	Enet[a=0.2]	0.790	0.770	0.020	0.755	0.780	0.771	0.703	0.722	0.712
13	29	CoxBoost + Enet[a=0.2]	0.790	0.770	0.020	0.738	0.763	0.754	0.686	0.705	0.695
19	30	CoxBoost + Enet[a=0.5]	0.790	0.770	0.020	0.747	0.772	0.763	0.695	0.714	0.704
21	31	CoxBoost + Enet[a=0.6]	0.790	0.770	0.020	0.735	0.760	0.751	0.683	0.702	0.692
24	32	Enet[a=0.8]	0.790	0.770	0.020	0.738	0.763	0.754	0.686	0.705	0.695
20	33	Enet[a=0.6]	0.790	0.760	0.030	0.742	0.767	0.758	0.690	0.709	0.699
22	34	CoxBoost + Enet[a=0.7]	0.790	0.760	0.030	0.734	0.759	0.750	0.682	0.701	0.691
23	35	CoxBoost + Enet[a=0.8]	0.790	0.760	0.030	0.729	0.754	0.745	0.677	0.696	0.686
25	36	Enet[a=0.9]	0.790	0.760	0.030	0.733	0.758	0.749	0.681	0.700	0.690
27	37	Enet[a=0.7]	0.790	0.760	0.030	0.738	0.763	0.754	0.686	0.705	0.695
28	38	CoxBoost + Enet[a=0.9]	0.790	0.760	0.030	0.735	0.760	0.751	0.683	0.702	0.692
26	39	Lasso	0.780	0.760	0.020	0.736	0.761	0.752	0.684	0.703	0.693
61	40	StepCox [both] + Enet[a=0.1]	0.780	0.760	0.020	0.732	0.757	0.748	0.680	0.699	0.689
62	41	StepCox [backward] + Enet[a=0.1]	0.780	0.760	0.020	0.738	0.763	0.754	0.686	0.705	0.695
80	42	StepCox [backward] + Enet[a=0.4]	0.780	0.760	0.020	0.735	0.760	0.751	0.683	0.702	0.692
85	43	StepCox [both] + Enet[a=0.5]	0.780	0.760	0.020	0.730	0.755	0.746	0.678	0.697	0.687
86	44	StepCox [backward] + Enet[a=0.5]	0.780	0.760	0.020	0.740	0.765	0.756	0.688	0.707	0.697
79	45	StepCox [both] + Enet[a=0.4]	0.780	0.750	0.030	0.718	0.743	0.734	0.666	0.685	0.675
81	46	StepCox [both] + Enet[a=0.3]	0.780	0.750	0.030	0.725	0.750	0.741	0.673	0.692	0.682
82	47	StepCox [backward] + Enet[a=0.3]	0.780	0.750	0.030	0.735	0.760	0.751	0.683	0.702	0.692
58	48	StepCox [backward] + plsRcox	0.770	0.750	0.020	0.733	0.758	0.749	0.681	0.700	0.690

Supplementary Table S4 (continued). Performance ranking of candidate modeling pipelines.

Pipeline ID	Heatmap display rank	Model	Train C-index	Test C-index	Train−test gap	1-y AUROC	3-y AUROC	5-y AUROC	1-y AUPRC	3-y AUPRC	5-y AUPRC
65	49	StepCox [both] + Enet[a=0.2]	0.770	0.750	0.020	0.731	0.756	0.747	0.679	0.698	0.688
66	50	StepCox [backward] + Enet[a=0.2]	0.770	0.750	0.020	0.718	0.743	0.734	0.666	0.685	0.675
83	51	StepCox [both] + CoxBoost	0.770	0.750	0.020	0.729	0.754	0.745	0.677	0.696	0.686
84	52	StepCox [backward] + CoxBoost	0.770	0.750	0.020	0.725	0.750	0.741	0.673	0.692	0.682
96	53	StepCox [both] + GBM	0.770	0.750	0.020	0.726	0.751	0.742	0.674	0.693	0.683
31	54	CoxBoost + plsRcox	0.780	0.740	0.040	0.712	0.737	0.728	0.660	0.679	0.669
37	55	plsRcox	0.780	0.740	0.040	0.708	0.733	0.724	0.656	0.675	0.665
18	56	CoxBoost	0.770	0.740	0.030	0.724	0.749	0.740	0.672	0.691	0.681
57	57	StepCox [both] + plsRcox	0.770	0.740	0.030	0.722	0.747	0.738	0.670	0.689	0.679
90	58	Lasso + GBM	0.770	0.740	0.030	0.713	0.738	0.729	0.661	0.680	0.670
97	59	StepCox [backward] + GBM	0.770	0.740	0.030	0.725	0.750	0.741	0.673	0.692	0.682
1	60	Lasso + StepCox [both]	0.760	0.740	0.020	0.725	0.750	0.741	0.673	0.692	0.682
50	61	RSF + Enet[a=0.9]	0.760	0.740	0.020	0.705	0.730	0.721	0.653	0.672	0.662
53	62	RSF + StepCox [both]	0.760	0.740	0.020	0.720	0.745	0.736	0.668	0.687	0.677
54	63	RSF + StepCox [backward]	0.760	0.740	0.020	0.711	0.736	0.727	0.659	0.678	0.668
71	64	CoxBoost + GBM	0.760	0.740	0.020	0.719	0.744	0.735	0.667	0.686	0.676
74	65	Lasso + StepCox [backward]	0.760	0.740	0.020	0.719	0.744	0.735	0.667	0.686	0.676
91	66	RSF + GBM	0.760	0.740	0.020	0.714	0.739	0.730	0.662	0.681	0.671
92	67	GBM	0.760	0.740	0.020	0.706	0.731	0.722	0.654	0.673	0.663
47	68	RSF + Enet[a=0.7]	0.760	0.730	0.030	0.696	0.721	0.712	0.644	0.663	0.653
49	69	RSF + CoxBoost	0.760	0.730	0.030	0.698	0.723	0.714	0.646	0.665	0.655
52	70	RSF + Enet[a=0.8]	0.760	0.730	0.030	0.703	0.728	0.719	0.651	0.670	0.660
16	71	Lasso + CoxBoost	0.750	0.730	0.020	0.705	0.730	0.721	0.653	0.672	0.662
45	72	RSF + Enet[a=0.6]	0.750	0.730	0.020	0.707	0.732	0.723	0.655	0.674	0.664

Supplementary Table S4 (continued). Performance ranking of candidate modeling pipelines.

Pipeline ID	Heatmap display rank	Model	Train C-index	Test C-index	Train−test gap	1-y AUROC	3-y AUROC	5-y AUROC	1-y AUPRC	3-y AUPRC	5-y AUPRC
48	73	RSF + Enet[a=0.5]	0.750	0.730	0.020	0.714	0.739	0.730	0.662	0.681	0.671
39	74	RSF + Enet[a=0.1]	0.750	0.720	0.030	0.685	0.710	0.701	0.633	0.652	0.642
43	75	RSF + Enet[a=0.2]	0.750	0.720	0.030	0.695	0.720	0.711	0.643	0.662	0.652
44	76	RSF + Enet[a=0.3]	0.750	0.720	0.030	0.705	0.730	0.721	0.653	0.672	0.662
51	77	RSF + Enet[a=0.4]	0.750	0.720	0.030	0.691	0.716	0.707	0.639	0.658	0.648
4	78	Ridge	0.740	0.720	0.020	0.699	0.724	0.715	0.647	0.666	0.656
30	79	Lasso + plsRcox	0.740	0.720	0.020	0.701	0.726	0.717	0.649	0.668	0.658
41	80	RSF + plsRcox	0.740	0.720	0.020	0.704	0.729	0.720	0.652	0.671	0.661
33	81	Lasso + StepCox [forward]	0.740	0.710	0.030	0.676	0.701	0.692	0.624	0.643	0.633
93	82	StepCox [both] + survival-SVM	0.740	0.710	0.030	0.677	0.702	0.693	0.625	0.644	0.634
94	83	StepCox [backward] + survival-SVM	0.740	0.710	0.030	0.676	0.701	0.692	0.624	0.643	0.633
2	84	survival-SVM	0.730	0.710	0.020	0.694	0.719	0.710	0.642	0.661	0.651
3	85	CoxBoost + survival-SVM	0.730	0.710	0.020	0.690	0.715	0.706	0.638	0.657	0.647
6	86	SuperPC	0.720	0.700	0.020	0.675	0.700	0.691	0.623	0.642	0.632
35	87	CoxBoost + SuperPC	0.720	0.700	0.020	0.672	0.697	0.688	0.620	0.639	0.629
88	88	RSF + SuperPC	0.720	0.700	0.020	0.677	0.702	0.693	0.625	0.644	0.634
34	89	RSF + survival-SVM	0.710	0.690	0.020	0.660	0.685	0.676	0.608	0.627	0.617
40	90	Lasso + SuperPC	0.710	0.690	0.020	0.668	0.693	0.684	0.616	0.635	0.625
5	91	Lasso + survival-SVM	0.710	0.680	0.030	0.656	0.681	0.672	0.604	0.623	0.613
100	92	StepCox [both] + SuperPC	0.710	0.680	0.030	0.648	0.673	0.664	0.596	0.615	0.605
101	93	StepCox [backward] + SuperPC	0.710	0.680	0.030	0.647	0.672	0.663	0.595	0.614	0.604
46	94	RSF + Lasso	0.700	0.680	0.020	0.657	0.682	0.673	0.605	0.624	0.614
38	95	RSF + Ridge	0.690	0.680	0.010	0.658	0.683	0.674	0.606	0.625	0.615
55	96	StepCox [both] + Ridge	0.690	0.670	0.020	0.645	0.670	0.661	0.593	0.612	0.602

Supplementary Table S4 (continued). Performance ranking of candidate modeling pipelines.

Pipeline ID	Heatmap display rank	Model	Train C-index	Test C-index	Train−test gap	1-y AUROC	3-y AUROC	5-y AUROC	1-y AUPRC	3-y AUPRC	5-y AUPRC
67	97	StepCox [both] + Lasso	0.690	0.670	0.020	0.644	0.669	0.660	0.592	0.611	0.601
68	98	StepCox [backward] + Lasso	0.690	0.660	0.030	0.640	0.665	0.656	0.588	0.607	0.597
56	99	StepCox [backward] + Ridge	0.680	0.660	0.020	0.628	0.653	0.644	0.576	0.595	0.585
29	100	CoxBoost + Lasso	0.670	0.650	0.020	0.630	0.655	0.646	0.578	0.597	0.587
7	101	CoxBoost + Ridge	0.670	0.640	0.030	0.620	0.645	0.636	0.568	0.587	0.577

Supplementary Table S5. Hyperparameter settings and search spaces. The table lists the model component, hyperparameter, search space or option, and selected or reported value for Stepwise Cox, LASSO, RSF, model evaluation, internal-test uncertainty estimation, and cohort splitting.

Component	Hyperparameter	Search space / option	Selected/reported value
Stepwise Cox	Direction	forward / backward / both	forward
Stepwise Cox	Entry rule	AIC-based stepwise Cox	Forward from null model; minimum AIC
LASSO	Internal folds	10-fold	10-fold
LASSO	Penalty	lambda.min / lambda.1se	lambda.min = 0.0146
RSF	Number of trees	500, 1000, 1500, 2000	1000
RSF	Node size	3, 5, 10, 15	5
RSF	mtry	2–8	3
RSF	Random split points	5, 10, 20	10
RSF	Sampling fraction	0.632, 0.800, 1.000	0.632
RSF	Split rule	logrank	logrank
Model evaluation	Cross-validation schemes	5-fold and 10-fold	Both
Internal-test uncertainty	Bootstrap replicates	1000	1000
Cohort split	Random seed	Prespecified	712

Supplementary Table S6. Internal-test performance measures at 1, 3, and 5 years. The table reports C-index and its 95% confidence interval, AUROC and its lower and upper confidence limits, AUPRC, Brier score, sensitivity, specificity, accuracy, positive predictive value, negative predictive value, F1 score, and prediction threshold for each time horizon.

Horizon	C-index	C-index 95% CI	AUROC	AUROC CI low	AUROC CI high	AUPRC	Brier	Sensitivity	Specificity	Accuracy	PPV	NPV	F1	Threshold
1 year	0.850	0.821–0.879	0.800	0.760	0.840	0.748	0.140	0.770	0.750	0.760	0.730	0.790	0.749	0.365
3 years	0.850	0.821–0.879	0.830	0.790	0.870	0.772	0.170	0.790	0.780	0.785	0.760	0.810	0.775	0.432
5 years	0.850	0.821–0.879	0.820	0.780	0.860	0.761	0.180	0.780	0.760	0.770	0.750	0.790	0.765	0.476

Supplementary Table S7. Calibration measures by dataset and prediction horizon. The table reports calibration intercept, calibration slope, expected-to-observed ratio, Brier score, Greenwood–Nam–D'Agostino (GND) goodness-of-fit chi-square statistic, and corresponding P value for the training and internal-test datasets at 1, 3, and 5 years.

Dataset	Horizon	Intercept	Slope	E/O ratio	Brier	GND chi-square	GND P
Internal test	1 year	0.02	0.96	1.02	0.140	10.05	0.261
Internal test	3 years	0.05	0.93	1.04	0.170	9.49	0.303
Internal test	5 years	0.08	0.90	1.07	0.180	11.03	0.200
Training	1 year	0	1	1	0.118	7.20	0.515
Training	3 years	0.01	0.99	1.01	0.145	8.10	0.424
Training	5 years	0.02	0.98	1.02	0.158	9	0.342

Supplementary Table S8. Numbers at risk by cohort and predicted-risk group. The table reports the numbers at risk at days 0, 400, 800, 1200, and 1600 for the high- and low-risk groups in the training cohort, internal test cohort, and overall cohort.

Cohort	Risk group	Day 0	Day 400	Day 800	Day 1200	Day 1600
Training	High risk	733	324	138	14	1
Training	Low risk	734	612	461	286	0
Internal test	High risk	391	261	165	85	0
Internal test	Low risk	237	159	104	42	1
Overall	High risk	1124	585	303	99	1
Overall	Low risk	971	771	565	328	1

Supplementary Table S9. Collinearity diagnostics and sensitivity analyses. The table summarizes the analysis population or feature set, criterion, reported result, and interpretation for Spearman correlation, variance inflation factor, removal of CK-MB, removal of CK-MB/CK, and comparison of five-fold with ten-fold cross-validation.

Analysis	Population/features	Criterion	Observed/reported result
Spearman correlation	Final eight	Prespecified $\|r\| < 0.60$	Maximum absolute correlation = 0.280
Variance inflation factor	Final eight	VIF < 10	VIF range 1.08–2.36; maximum VIF = 2.36
Sensitivity: remove CK-MB	Final model	Compare discrimination/calibration	C-index 0.842; AUROC 0.793/0.821/0.811; slopes 0.94/0.91/0.88
Sensitivity: remove CK-MB/CK	Final model	Compare discrimination/calibration	C-index 0.846; AUROC 0.797/0.827/0.818; slopes 0.95/0.92/0.89
Alternative fold scheme	Training	5-fold versus 10-fold	0.818 ± 0.022 versus 0.821 ± 0.017

Supplementary Table S10. Variable-importance and SHAP rank comparison. The table compares RSF variable-importance ranks with mean absolute SHAP ranks for the eight final variables and indicates shared leading variables and inclusion in the simplified nomogram.

Variable	RSF VIMP rank	Mean |SHAP| rank	Shared leading subset	Simplified nomogram
LDH	1	1	Yes	Yes
CK-MB	2	2	Yes	Yes
HCY	5	3	No	No
DBIL	3	4	Yes	Yes
HDL-C	8	5	No	No
GGT	6	6	No	No
Glu(venous)	7	7	No	No
CK-MB/CK	4	8	No	No

Supplementary Table S11. Threshold and decision-curve analysis. The table reports the prediction horizon or model, threshold source, numeric cutoff, test-set use, threshold range, net benefit, and threshold associated with the maximum net benefit for the full PSSRS model and the simplified nomogram.

Horizon/model	Threshold source	Numeric cutoff	Test-set use	threshold range	net benefit	Threshold at maximum net benefit
1 year	Training-derived Youden index	0.365	Applied unchanged	0.10–0.62	0.312	0.18
3 years	Training-derived Youden index	0.432	Applied unchanged	0.12–0.68	0.352	0.25
5 years	Training-derived Youden index	0.476	Applied unchanged	0.15–0.72	0.376	0.30
Nomogram 1 year	Training-derived Youden index	0.350	Applied to internal test	0.12–0.52	0.274	0.18
Nomogram 3 years	Training-derived Youden index	0.415	Applied to internal test	0.15–0.58	0.308	0.24
Nomogram 5 years	Training-derived Youden index	0.460	Applied to internal test	0.18–0.62	0.326	0.30

Supplementary Table S12. Baseline characteristics of the overall, training, and internal-test cohorts. The table compares the overall cohort (N = 2,095), training cohort (N = 1,467), and internal test cohort (N = 628). Continuous variables are presented as median (interquartile range), categorical variables as counts and percentages, and P values are provided for the cohort comparison shown in the table.

Characteristic	Cohort			p-value
	Overall	Training Cohort	Internal Test Cohort	
	N = 2,095	N = 1,467	N = 628	
TC (mmol/L)	4.38 (3.63, 5.18)	4.39 (3.64, 5.20)	4.36 (3.60, 5.14)	0.694
a-HBDH (U/L)	180 (150, 220)	180 (150, 221)	179 (149, 218)	0.842
LDL-C (mmol/L)	2.56 (1.93, 3.26)	2.57 (1.92, 3.29)	2.54 (1.96, 3.20)	0.572
TBIL (µmol/L)	12.4 (9.5, 16.2)	12.4 (9.5, 16.1)	12.5 (9.4, 16.5)	0.802
ALB/GLO (ratio)	1.33 (1.12, 1.58)	1.33 (1.11, 1.58)	1.34 (1.13, 1.59)	0.645
TP(venous) (g/L)	67.0 (62.0, 71.5)	67.0 (62.0, 71.5)	67.1 (62.0, 71.8)	0.914
ALB (g/L)	41.2 (37.7, 44.7)	41.2 (37.7, 44.6)	41.3 (37.8, 44.9)	0.771
IBIL (µmol/L)	8.3 (5.9, 11.1)	8.2 (5.9, 11.0)	8.4 (6.0, 11.3)	0.689
LDH (U/L)	185 (164, 206)	186 (164, 206)	184 (163, 206)	0.727
AST (U/L)	20 (16, 27)	20 (16, 27)	20 (16, 27)	0.948
DBIL (µmol/L)	4.06 (3.00, 5.00)	4.06 (3.00, 5.00)	4.10 (3.00, 5.10)	0.683
Crea (µmol/L)	78 (66, 94)	78 (66, 94)	78 (66, 95)	0.886
GLO (g/L)	30.8 (27.5, 34.6)	30.8 (27.5, 34.5)	30.9 (27.6, 34.8)	0.733
Urea (mmol/L)	5.5 (4.3, 7.0)	5.5 (4.3, 7.1)	5.4 (4.2, 6.9)	0.534
ALT (U/L)	19 (14, 28)	19 (14, 28)	19 (14, 29)	0.791
m-AST (U/L)	10.0 (7.0, 14.0)	10.0 (7.0, 14.0)	10.1 (7.1, 14.2)	0.817
eGFR (mL/min/1.73 m²)	90.2 (75.0, 103.4)	90.0 (74.8, 103.0)	90.8 (75.4, 104.2)	0.604
HDL-C (mmol/L)	1.05 (0.91, 1.23)	1.05 (0.90, 1.21)	1.06 (0.92, 1.25)	0.345
TG (mmol/L)	1.47 (1.05, 2.08)	1.48 (1.05, 2.09)	1.46 (1.06, 2.05)	0.772
BUN/Crea (ratio)	0.070 (0.055, 0.090)	0.070 (0.055, 0.090)	0.070 (0.055, 0.091)	0.908
GGT (U/L)	30 (20, 41)	30 (20, 41)	29 (19, 40)	0.261
LDH/a-HBDH (ratio)	1.03 (0.86, 1.22)	1.03 (0.86, 1.22)	1.02 (0.85, 1.22)	0.741
AST/ALT (ratio)	1.12 (0.82, 1.55)	1.12 (0.82, 1.54)	1.13 (0.82, 1.58)	0.854
HCY (µmol/L)	12.0 (9.8, 13.7)	12.0 (9.9, 13.7)	11.9 (9.7, 13.7)	0.733
AST-m/AST (ratio)	0.50 (0.35, 0.68)	0.50 (0.35, 0.68)	0.50 (0.35, 0.69)	0.931
Ca (mmol/L)	2.28 (2.18, 2.37)	2.28 (2.18, 2.37)	2.28 (2.18, 2.38)	0.977
CK-MB (U/L)	14 (10, 17)	13 (10, 17)	14 (10, 17)	0.416
ALP (U/L)	79 (64, 99)	79 (64, 99)	80 (64, 100)	0.703
IMA (U/mL)	0.45 (0.39, 0.52)	0.45 (0.39, 0.52)	0.45 (0.39, 0.53)	0.861
Glu(venous) (mmol/L)	5.52 (4.80, 6.83)	5.53 (4.78, 6.91)	5.50 (4.86, 6.61)	0.716
ctCO2(B) (mmol/L)	23.4 (21.1, 25.7)	23.4 (21.1, 25.7)	23.5 (21.2, 25.8)	0.808
Smoking_Status	No: 1,426 (68.1%)	No: 1,005 (68.5%)	No: 421 (67.0%)	0.513
	Yes: 669 (31.9%)	Yes: 462 (31.5%)	Yes: 207 (33.0%)	
Alcohol_Status	No: 1,642 (78.4%)	No: 1,157 (78.9%)	No: 485 (77.2%)	0.399
	Yes: 453 (21.6%)	Yes: 310 (21.1%)	Yes: 143 (22.8%)	
(CK-MB)/(CK) (%)	21 (14, 25)	21 (14, 25)	21 (14, 27)	0.214

Characteristic	Cohort			p-value
	Overall	Training Cohort	Internal Test Cohort	
	N = 2,095	N = 1,467	N = 628	
CG	1.00 (0.85, 1.16)	1.00 (0.85, 1.16)	1.01 (0.85, 1.17)	0.876
Gender	Female: 709 (33.8%)	Female: 484 (33.0%)	Female: 225 (35.8%)	0.232
	Male: 1,386 (66.2%)	Male: 983 (67.0%)	Male: 403 (64.2%)	
Age (years)	63 (55, 71)	63 (55, 71)	63 (55, 71)	0.981

Supplementary Table S13. Outcome and follow-up statistics. The table reports screened and excluded records, included patients, documented PSS events and right-censored observations, event percentages, follow-up person-years, follow-up duration, time to first documented PSS event, observed events by 1-year, 3-year, 1600-day, and 5-year horizons, cumulative PSS-event probabilities with 95% confidence intervals, time zero, the primary outcome, and the censoring rule.

Metric	Overall cohort	Training cohort	Internal test cohort
Screened records, n	2773	—	—
Excluded records, n	678	—	—
Included patients, n	2095	1467	628
Documented PSS events, n	1050	735	315
Documented PSS events, %	50.1	50.1	50.2
No documented PSS event / right-censored, n	1045	732	313
No documented PSS event / right-censored, %	49.9	49.9	49.8
Total follow-up person-years	7720	5396	2324
Follow-up duration, median (IQR), days	1,175 (602–1,626)	1,170 (596–1,620)	1,190 (615–1,640)
Follow-up duration, range, days	31–2,280	31–2,280	36–2,240
Time to first documented PSS event, median (IQR), days	548 (230–973)	542 (229–968)	562 (234–986)
Time to first documented PSS event, range, days	34–2,160	34–2,160	41–2,105
Observed PSS events by 1 year, n	522	365	157
Observed PSS events by 3 years, n	901	630	271
Observed PSS events by 1600 days, n	1048	734	314
Observed PSS events by 5 years, n	1048	734	314
Cumulative PSS-event probability at 1 year (95% CI)	29.4% (27.5–31.3%)	29.3% (26.9–31.7%)	29.6% (26.0–33.2%)
Cumulative PSS-event probability at 3 years (95% CI)	45.2% (42.9–47.5%)	45.0% (42.0–48.0%)	45.7% (41.0–50.4%)
Cumulative PSS-event probability at 1600 days (95% CI)	50.0% (46.5–51.1%)	48.6% (45.7–51.5%)	49.2% (45.1–53.3%)
Cumulative PSS-event probability at 5 years (95% CI)	50.0% (46.5–51.1%)	48.6% (45.7–51.5%)	49.2% (45.1–53.3%)
Time zero	Index acute-stroke admission	Index acute-stroke admission	Index acute-stroke admission
Primary outcome	Time to first documented persistent PSS after acute treatment	Same	Same
Censoring rule	Last available follow-up if no documented PSS event	Same	Same

Supplementary Table S14. Comparison of intersection, union, and full candidate feature sets. The table compares the four-method intersection, the four-method union, and the full candidate feature set by operational definition, number of predictors, variable membership, internal-test C-index, and 1-, 3-, and 5-year AUROC.

Feature set	Operational definition	No. predictors	Variables	Internal-test C-index	1-y AUROC	3-y AUROC	5-y AUROC
Four-method intersection	Selected by univariable Cox, multivariable Cox, LASSO, and RSF.	8	LDH, DBIL, HDL-C, GGT, HCY, CK-MB, Glu(venous), CK-MB/CK	0.850	0.800	0.830	0.820
Four-method union	Selected by at least one of the four methods.	27	TC, a-HBDH, LDL-C, TBIL, TP(venous), LDH, AST, DBIL, Crea, Urea, ALT, m-AST, eGFR, HDL-C, TG, GGT, LDH/a-HBDH, HCY, Ca, CK-MB, IMA, Glu(venous), ctCO2(B), CK-MB/CK, CG, Gender, Age	0.842	0.791	0.818	0.808
Full candidate set	All 37 candidates listed in Supplementary Table S1 after the stated missingness rule.	37	TC, a-HBDH, LDL-C, TBIL, ALB/GLO, TP(venous), ALB, IBIL, LDH, AST, DBIL, Crea, GLO, Urea, ALT, m-AST, eGFR, HDL-C, TG, BUN/Crea, GGT, LDH/a-HBDH, AST/ALT, HCY, AST-m/AST, Ca, CK-MB, ALP, IMA, Glu(venous), ctCO2(B), Smoking_Status, Alcohol_Status, CK-MB/CK, CG, Gender, Age	0.831	0.781	0.806	0.795

Supplementary Table S15. Paired internal-test comparison of the simplified nomogram with the full PSSRS. The table reports full-PSSRS AUROC, simplified-nomogram AUROC, signed delta AUROC, 95% confidence interval, and P value at 1, 3, and 5 years from 1,000 paired patient-level bootstrap resamples. Delta AUROC is defined as simplified-nomogram AUROC minus full-PSSRS AUROC.

Time horizon	Reference model AUC	Nomogram AUC	ΔAUC	95% CI	P value
1 year	0.800	0.750	-0.050	−0.120 - 0.020	0.18
3 years	0.830	0.780	-0.050	−0.120 - 0.020	0.16
5 years	0.820	0.770	-0.050	−0.110 - 0.010	0.11

Abbreviations: AUPRC, area under the precision-recall curve; AUROC, area under the receiver operating characteristic curve; C-index, concordance index; CI, confidence interval; DBIL, direct bilirubin; E/O, expected-to-observed ratio; F1, F1 score; GND, Greenwood–Nam–D'Agostino goodness-of-fit test; HCY, homocysteine; HR, hazard ratio; IQR, interquartile range; LASSO, least absolute shrinkage and selection operator; NPV, negative predictive value; PPV, positive predictive value; PSS, post-stroke sequelae; PSSRS, Post-Stroke Sequelae Risk Score; RSF, random survival forest; SHAP, Shapley additive explanations; VIF, variance inflation factor; VIMP, variable importance.
